# Adaptive Disorder as the Hallmark of Nanobodies Antigen-Binding Loops

**DOI:** 10.1021/acs.jcim.6c00716

**Published:** 2026-05-22

**Authors:** Davide Bagordo, Gauthier Trèves, Mariangela Santorsola, Giorgio Colombo, Francesco Lescai

**Affiliations:** † Department of Biology and Biotechnology “L. Spallanzani”, 19001 University of Pavia, Pavia 27100, Italy; ‡ Department of Chemistry, University of Pavia, Pavia 27100, Italy

## Abstract

Nanobodies are antigen-binding proteins of great interest as diagnostics and therapeutics. Accurate and fast characterization of their complementarity-determining regions (CDRs) is crucial to uncover the principles guiding their design. Yet, this task remains challenging, as random recombination and somatic mutations generate highly diverse CDR sequences that escape motif-based or structure-prediction approaches currently used to identify them. To overcome this hurdle, we employed two independent strategies that converged on the same conclusion. At the sequence level, we developed a deep learning model to identify nanobody CDRs directly from the primary sequence. At the structural level, we applied an energy decomposition method, revealing CDRs as residues highly uncoupled to the rest of the fold. Explainability analyses showed the network captured intrinsic CDR properties, which notably aligned with these energy values. CDRs emerge as fuzzy regions capable of adopting diverse conformational ensembles, from which a preferred state is selected upon antigen binding. This finding supports a model where chaos in both sequence and structure appears adaptive and disorder emerges as the hallmark of nanobody CDRs. This work aims to advance the definition of rules for the design of antigen binding regions, paving the way for the next-generation immune diagnostics and therapeutics.

## Graphical abstract

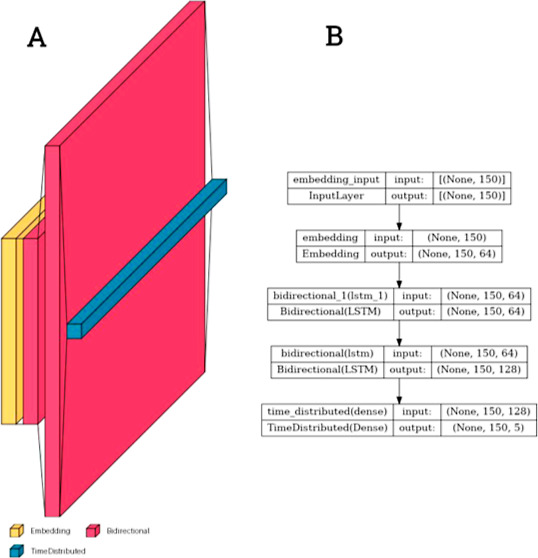

## Introduction

Protein–protein interactions (PPIs) are fundamental determinants of virtually all biological processes. Within the immune system, precise molecular recognition between host proteins and pathogenic antigens initiates cascades of biochemical events that ultimately drive the clearance of invading agents. Deciphering the sequence and structural principles that underlie these interactions is therefore central to understanding the mechanisms of immune defense.

From a conceptual standpoint, insights into the evolutionary dynamics of sequences and the molecular origins of immunological recognition can illuminate why particular biomolecules are selected as effective mediators and define the structural features that govern their binding interfaces. From a translational perspective, such knowledge provides a framework for establishing molecular design rules, thereby advancing the development of next-generation biologics—a field that has gained remarkable momentum in recent years.

In this context, Nanobodies (Nbs) have been attracting growing interest. Nbs are a class of antigen-binding fragments naturally present in camelid and shark serum.^1^ They are also called V_H_H antibodies, since they are constituted only of the antibody heavy chain. They show the same architecture as antibody V_H_ domains, with four conserved framework regions (FR 1/2/3/4) around three hypervariable loops, called complementarity-determining regions (CDR 1/2/3). CDRs, along with a small number of framework residues, form the paratope region responsible for the interaction with the antigen epitope. Despite the involvement of all three loops, the main actor in epitope recognition and binding is CDR3. This region is much longer than the corresponding one in conventional antibodies, and it often adopts long fingerlike conformations able to interact inside cavities formed on antigens. Consequently, epitopes targeted by Nbs are often different from those targeted by conventional antibodies.^2^

Moreover, the smaller size of nanobodies as well as the different amino acidic composition results in numerous advantages: a higher stability and water solubility, and a stronger binding affinity for the antigen.^3^ These unique features make Nbs very attractive as next-generation biodrugs, as it is also confirmed by the sharp increase in Nbs intended for cancer diagnosis and treatment.^4^

In the last five years, the development of Nbs was strongly enhanced by the Sars-CoV-2 global pandemic. A wide number of Nbs were indeed produced as both diagnostic and therapeutic tools to face the global threat. Different targets were identified, the main one being the Receptor Binding Domain (RBD) of the Spike glycoprotein. Indeed, during the infection onset, Sars-CoV-2 binds to the angiotensin converting enzyme 2 (ACE2) using the Receptor Binding Site (RBS), a subdomain of RBD.^5^ Most Nbs developed to neutralize Sars-CoV-2 compete, partially or completely, with ACE2 for the binding to RBS.

The development of both Nbs and antibodies (Abs) is however time-consuming and expensive. In silico methods^6^ have gained an increasingly relevant role in this process. Machine Learning (ML) and Deep Learning (DL) methods have been developed for the generation of sequences and for 3D structure prediction of Immunoglobulins (Igs), which entails both Abs and Nbs. However, Igs are more challenging to handle compared to other proteins, since CDRs, and CDR3 in particular, do not have an evolutionary conserved sequence which can guide the definition of rules and patterns for generative or predictive tools. This is because their sequences are generated through the *V­(D)­J* recombination occurring during the development of B and T cells, as well as through somatic mutations occurring during the so-called affinity maturation. This entirely random generative mechanism is responsible for the extremely diverse repertoire of CDR3 sequences^7^ which allows the immune system to recognize an incredibly diverse array of antigens. Identifying and characterizing the complementarity-determining regions is therefore the key step in describing an immune repertoire, either in vivo or during the synthesis of immuno-therapies.

The state-of-the-art methods to identify CDRs are based on similarities among immunoglobulin structures and sequences: some of the most widely adopted are Kabat,^8^ Chothia,^9^ AbM^10^ and IMGT^11^ numbering schemes. Each of these schemes identify CDRs from the sequence by searching for different recurrent amino acidic motifs. The fact that CDRs are generated randomly and only later selected based on their affinity with antigens represents however a massive difference compared to other sequence motifs such as transcription factor DNA binding sites or protein family domains.

For these reasons, motif-based search methods applied to CDRs will inevitably show limitations, which are alleviated by successive updates of the definition rules as new molecules emerge. Therefore, even though these schemes are largely reliable and widely used, inaccuracies, particularly in nanobodies, could potentially lead to confusion and discrepancies in the definition of CDRs,^12^ and particularly CDR3. Structure prediction methods for Igs pose an even tougher challenge, given the peculiar malleability and conformational diversity of CDRs. Although tools such as AlphaFold3 (AF3) have enormously enhanced our capability to predict protein structures, they still have difficulties in predicting CDR3 conformations. Even though CDR3 loop prediction improves when the antigen is given, AF3 still has a 60% failure rate in Abs and Nbs docking with their target when using a single seed sampling.^13^ In summary, the intrinsic diversity of CDR3 sequences and conformations hampers both the structure prediction and the docking precision.

Here, we asked whether we can harness the observed diversity/randomness in sequence generation and the apparent tendency for flexibility/disorder in immunoglobulin recognition regions to define a general, unbiased framework for CDR(3) identification. By employing a multidisciplinary approach, we set out to address this key challenge using Nbs elicited against SARS-CoV-2 RBD as test cases.

To proceed along this path, we make use of the general concept of fuzziness and fuzzy regions in the context of biomolecular interactions. From the sequence point of view, fuzziness in binding regions could aptly indicate a tendency for efficient exploration of the sequence space, free of constraints related e.g. to fold stabilization. From the structural perspective, fuzziness can favor adaptability to target partners via conformational selection mechanisms. This aspect has been associated with the function of intrinsically disordered regions (IDRs) involved in protein–protein interactions,^15^ and has been increasingly recognized as a key structural driver in antibody–antigen recognition.^17,18^

We overcome motif-based or structure-prediction based methods for characterizing the repertoire, which do not address the intrinsic biological characteristics of CDRs, and provide a straightforward method, based on deep-learning, to predict CDRs solely based on the Nb sequences. In parallel, we investigate the possibility to unbiasedly isolate a subset of surface residues from Nbs structures and recognize them as potential CDRs by analyzing their intraprotein energetic properties in the isolated, unbound Nb. Via structure-based energy decomposition, we indeed show that CDR3s displays a peculiar intramolecular interaction profile consistent with high intrinsic flexibility and consequent adaptability.

Taken together, our results define hypervariable loops as intrinsically disordered regions, indicating that conformational fuzziness underlies immunoglobulin polyreactivity, and show that independent sequence- and structure-based approaches converge on the same principle: explainable-AI methods reveal that the features learned by neural networks mirror those identified through interaction energy analyses, namely the intrinsically fuzzy nature of CDRs.

## Materials and Methods

### Development of a Deep Learning Architecture

The model architecture has been developed and trained using a Docker container environment for reproducibility, with Python 3.11.0rc1 on linux and Tensorflow version 2.15.1, Keras version 2.15.0 running on hardware with NVIDIA-SMI 550.90.07, driver version 550.90.07 and CUDA version 12.4.

### Encoding and Decoding

The prediction of CDR sequences from a nanobody sequence has been designed as a classification task, i.e. predicting whether each residue in the sequence belonged to CDR1, CDR2, CDR3 or the rest of the protein (which we called “body”). Based on the annotated CDRs therefore, a script has been designed to search for each of the CDR sequences within the nanobody and convert each residue on its corresponding class, to be used as a label for the training of a supervised classification neural network. Body has been assigned class 0, CDR1 class 1, CDR2 class 2, CDR3 class 3. Additionally, to create a consistent data set with equal-length inputs, each nanobody sequence has been padded to 150-residues length and padded null residues have been assigned class 4 (with a weight 0, for the training of the network). This scheme allows us to encode and decode any nanobody sequence and use the model predictions (see below) to extract the specific residues (sequence) of any CDR of interest in the nanobody sequence. As far as the input sequences are concerned, we simply assigned a sequential integer token to each amino acid residue, thus building a simple vocabulary of fixed size corresponding to all possible residues, plus a null-value residue for the padded regions.

## Architecture

Based on the encoding of choice, and the need to capture the context of the residues flanking each CDR on both sides, we decided to employ a sequential architecture tailored for sequence labeling tasks. The first layer of the network is an embedding layer (with dimension = 64), which serves the purpose of learning dense vector representations of the tokens from the above-described vocabulary, with a maximum input sequence length of 150 tokens. Following the embedding layer, we added two stacked bidirectional long short-term memory (BiLSTM) layers, with 32 and 64 units respectively, both configured to return full sequences in order to preserve the temporal context across the two layers. According to our choice regarding the encoding of the labels, the output layer consists of a time-distributed dense layer, with a softmax activation function, yielding class probabilities at each time step across five categorical outputs (our categories), i.e. including the padding class. The model was trained using the Adam optimizer, and we imposed a learning rate of 1*10–4 and a gradient clipping (value 1.0), meant to mitigate potentially exploding gradients, when handling a large data set encompassing millions of sequences. We used sparse categorical crossentropy as the loss function, and accuracy was computed comparing each residue on each nanobody sequence, with a weighting designed to ignore padding tokens (Supporting Information, 1.2). The total number of parameters in the model is 92.869, all of them trainable.

### Training and Validation Data

The Integrated Nanobody Database for Immunoinformatics (INDI)^19^ is a comprehensive repository aggregating camelid single-domain antibody (VHH) sequences from five public sources: the Protein Data Bank (PDB)^20^ for structural chains, patent literature, NCBI GenBank records, high-throughput next generation sequencing (NGS) data sets and peer-reviewed publications. Each INDI entry consists of a complete VHH sequence linked to metadata from its original database. Raw sequences were filtered to obtain full-length VHH domains containing all three canonical CDR loops and comprising only the 20 standard amino acids. All sequences are annotated using an IMGT-based numbering scheme.^11^ INDI comprises more than 11 million nanobody sequences. It provides the data in two linked flat-files (FASTA for sequences and tab-delimited metadata tables). This curated INDI data set was used as the training corpus for our deep-learning CDR prediction model.

We split the data into 80% of the sequences used for training, and 20% of the sequences used for validation using a standard random split. We acknowledge that the intrinsic redundancy of NGS data within INDI inherently introduces a degree of sequence similarity between these two sets. In order to process such a massive data set efficiently, data has been chunked in tensors of 100,000 sequences each (i.e., shape [100,000, 150]), each saved as a separate file on disk. During training, data were loaded by chunk and a shuffling approach within batches (size 1024) was applied to control memory usage and improve generalization. The training chunks were 90 containing a total of 8,982,880 nanobody sequences, while the validation chunks were 23, containing a total of 2,245,720 sequences. The total number of sequences used to build the model was therefore 11,228,600. We have trained the model for 4 epochs, each loading the data by chunk and limiting the batch size to 1024 to control the memory usage.

### Testing Data Set

In this work, we selected a data set of 3D structures of 121 nanobodies (Nbs) known to bind the Receptor Binding Domain (RBD) of the SARS-CoV-2 Spike glycoprotein. The Nbs were selected by downloading them from the Protein Data Bank,^20^ and crossing information with that available in CoV-AbDab,^21^ a Sars-CoV-2 dedicated database, and in SabDab-nano,^22^ a nanobody centered repository. We extracted Nbs with corresponding 3D structures solved by X-ray Diffraction Crystallography or Electron Microscopy, to generate a list of 121 nonredundant and monovalent biomolecules. Their respective PDB codes are reported in Supporting Information, 3.1.

A second independent data set was constructed by selecting 7 new sequences (Supporting Information, 3.2) from the INDI database, specifically chosen as representatives of highly divergent clusters. Their 3D structure was modeled using the AlphaFold3 Server.^14^

### Computing Infrastructure

The training of the model has been conducted using the Google Cloud Computing Platform. We employ Terraform^23^ to create reproducible infrastructure as-a-code (IaaC), and standardize our operations. To train the deep learning architecture on the INDI data, we configured a “a2-highgpu-1g” virtual machine, equipped with 12 vCPUs and 85GB RAM and a local SSD along with an NVIDIA Tesla A100 GPU with 40GB memory.

### Nanobody Characterization

#### CDRs Identification on Nbs Sequences Using Standard Numbering Schemes

The Multiple Sequence Viewer/Editor tool from the Maestro Schrödinger suite version 2024-3 (Schrödinger Release 2024-3: Maestro, Schrödinger, LLC, New York, NY, 2024.) was employed to identify CDRs on Nbs by using the Chothia annotation scheme.^9^ After anchor residues are found and alignment to the reference is performed, the CDRs are annotated with the following scheme: CDR1: positions 26–32; CDR2: positions 53 to 55; CDR3: positions 96 to 101 (insertions with lettered positions are allowed to account for longer CDRs).^9^ CDRs identified in this way are considered as the ground truth reference. To systematically evaluate the robustness of our model against multiple established definitions, we expanded our ground-truth annotations to include the IMGT^11^ and Martin^10^ numbering schemes. This step was performed programmatically using the ANARCI^24^ (Antigen receptor numbering and receptor classification) tool, deployed via a dedicated Conda environment (Python 3.10).

Nanobody sequences were processed using the IMGT numbering scheme in ANARCI. The CDR sequences were then extracted strictly following the standard IMGT Scientific Chart boundaries (CDR1: positions 27–38, CDR2:56–65, CDR3:105–117).^11^

Because AbM definitions rely on Chothia-based positioning, the sequences were first numbered using the Chothia scheme in ANARCI. Subsequently, the standard AbM ranges were applied to extract the relevant loops (CDR1: positions 26–35, CDR2:50–58, CDR3:95–102).^10^

### Energy Calculation

#### Structure Preparation

The 121 selected Nbs structures downloaded from the PDB were first visually inspected via the Pymol molecular modeling package (The PyMOL Molecular Graphics System, Version 3.0 Schrödinger, LLC). Selected Nbs were extracted from the complex with RBD and crystallographic waters were removed.

The structures were then prepared for the energy calculation. First, residues missing from the original PDB structures were added. The Protein Linker Design tool from the Maestro Schrödinger suite version 2024-3 (Schrödinger Release 2024-3: Maestro, Schrödinger, LLC, New York, NY, 2024.) was used to reconstruct unsolved loops in five Nbs (7C8W_MR17, residues W111 and G112; 7CAN_MR17-K99Y, residues Y108, D109, Y110, W111 and G112; 7KN5_VHHU, residues G10 and G66; 7N9E_Nb34, residues D101, P102, Y103 and G104; 8BEV_W25, residues S55 and H56). The newly added residues form a linker between two attachment points. The *Interdomain link library* method was selected to predict linker conformations through a database of interdomain linkers from the PDB. The strain energy of the new loop was calculated in implicit solvent using *Prim*e^25^ with the OPLS4 force field,^26^ and it represents the difference between the linker in the minimized conformation adopted in the protein and in its free isolated minimized conformation. The structure of the loop within the protein which maximizes the difference to the free isolated conformation, representing the minimum energy of the loop conformation in the protein, is selected as the representative conformation.

Next, each of the 121 Nbs underwent the Protein Preparation Workflow^27^ tool of the Maestro Schrödinger suite version 2024-3 (Schrödinger Release 2024-3: Maestro, Schrödinger, LLC, New York, NY, 2024.). Proteins were correctly protonated at pH of 7.4 and H-bonds assignments were optimized using PROPKA,^28^ side chains missing from the original PDB were added and disulfide bonds between Cys residues with their respective terminal thiol groups within 3.2 Å were reconstructed. In the last step, Nbs structures were minimized using OPLS4 force field^26^ with the implicit solvent model implemented in Maestro.

### Energy Decomposition Method for the Prediction of Potential Interacting Regions (CDRs) on Nanobodies

The structures of the Nbs prepared as described above were subsequently converted into an Amber-compliant PDB file using the *pdb* 4amb *er* utility included in the *Amber24 suite*.^29^

Next, each Nb structure was used as input for the structure- and interaction energy-based prediction of potential interacting regions using the Matrix of Low Coupling Energies (MLCE) method.^30^

The scripts and instructions to run this analysis are available on GitHub (https://github.com/colombolab/MLCE). Proteins are prepared and analyzed using the AmberTools suite^29^ with the ff14SB force field,^33^ following the steps described below.

First, each Nb PDB is converted to the AMBER format by using the *tleap* utility, obtaining a topology file and a configuration file (respectively in *.prmtop* and *.inpcrd* format). Next, the system is minimized in implicit solvent through 200 steps of steepest descent minimization using the *sander* executable.

In the third step, the nonbonded part of the potential, including van der Waals, electrostatic interactions and solvent effects, is computed via an MM/GBSA calculation performed with AmberTools python script *MMpbsa.pl*. For a Nb of *N* residues, the output is a *NxN* symmetric interaction matrix *M*_*ij*_, in which only van der Waals and electrostatic terms are summed

1 $$M_{ij}=E_{ij}^{vdW}+E_{ij}^{coul}$$

The original Energy Matrix can be diagonalized and reconstructed using the resulting eigenvalues and eigenvectors

2 $$M_{ij}=\sum_{k=1}^{N}\lambda_{k}v_{i}^{k}v_{j}^{k}$$

where λ_*k*_ is the *k*th eigenvalue, and *v*_*i*_^*k*^ is the *i*th component of the corresponding eigenvector. The eigenvectors are then labeled from the most negative to the most positive. We previously showed^30^ that it can be assumed that the first (most negative) eigenvalue, labeled λ_1_, and the associated eigenvector, namely *v*_1_, contain most of the information on the stabilizing interactions of the system. An approximated interaction matrix *M̃*_*ij*_ can thus be defined as

3 $$\tilde{M_{ij}}=\lambda_{1}v_{i1}v_{j1}$$

As the 3D structure of the protein under exam is known, for each Nb a contact matrix *C*_*ij*_ can be built by considering two amino acids in contact if the distance between two of their heavy atoms is smaller than 6 Å threshold. The Hadamard product of the two matrices gives us the matrix of the local coupling energies, i.e. information on the pairs of residues in contact in the 3D structure and the energetic intensity of this coupling.

4 $$MLCE_{ij}=\tilde{M_{ij}}\cdot C_{ij}$$

Potential interaction substructures are identified as sets of close-by residues that show weakest coupling interactions with the rest of the protein. Regions of weak coupling, also called patches, are thus identified by setting a percentage threshold. In this work, a 10% threshold was used, meaning that patches are composed of residues involved in the 10% of interactions that are less favorable energetically.

In this framework, regions of weak coupling with the rest of the protein also coincide with substructures that are generally prone to undergo substantial conformational changes, tolerate mutations without impacting on the overall 3D organization of the cognate biomolecule, and adapt to a binding partner with minimal energetic expense. This approach was validated in multiple instances against experimental data.^31^ The aforementioned properties can also be considered hallmarks of fuzziness for putatively interacting regions.

In the case of Nbs, it can be aptly hypothesized that the regions that emerge as most prone to interaction may in fact coincide with Complementarity Determining Regions (CDRs). Supporting Information, 3.1 shows that patches identified on Nbs include residues of CDRs in most cases (residues are always numbered from 1, with residue 1 being the first resolved residue in the crystal structure).

### Explainability

On the trained model used for inference on the RCSB data set, different methods have been used to explain the predictions, described in the following paragraphs.^35^

### Saliency Analysis

Gradient-based saliency maps were computed to assess the relative importance of each input position for model predictions across sequence timesteps. For each input sequence in inference mode, we identified the predicted class at each output time step and then computed the gradient of the corresponding class probability with respect to the embedding output. This has been done using TensorFlow’s automatic differentiation (*tf.GradientTape*). The model was functionally decomposed into its embedding layer and downstream recurrent layers, enabling position-wise gradient analysis. At each time step *t*, the absolute gradient values across embedding dimensions were averaged to yield a saliency score for each input position *p*, resulting in a full saliency matrix indexed by both time step and position. These values were initially recorded as individual records for every (*t*, *p*) pair. To facilitate interpretability, we then aggregated the saliency values by averaging across *t*, producing a summary measure that quantifies the average importance of each residue *p* in predicting class *c* at any position in the sequence. This final representation allows position-wise comparison of contributions to model decisions independent of output location.^37^ We used these values to produce saliency plots for each of the predicted classes along each nanobody sequence.

For a quantitative comparative analysis, residues of all nanobodies in the testing data set were further stratified into “peak” regions (top 20% of the saliency distribution matching the predicted class) and “trough” regions (lower 80% outside the target prediction), and their corresponding energy values were compared using a Wilcoxon rank-sum test.^39^

### Hidden States Analysis

Hidden state vectors from the final bidirectional LSTM were extracted for all nonpadding residues across all input sequences of the RCSB data set described above. The trained model was decomposed to expose the intermediate activations: this was done by assembling a submodel from the original input and through the embedding and following recurrent layers, but terminating prior to the classification output. For each sequence we then computed the full sequence of hidden states and collected those corresponding to nonpadding positions. Hidden states were stored alongside their metadata including the input position, predicted class at that position, token identity and sequence identifier in order to facilitate downstream analysis.^41^

Principal component analysis was then applied to the matrix of hidden state vectors, after normalizing all numeric features. PCA was performed using the *tidymodels* package, and projecting the data on 2 principal components to facilitate visual representation. We then joined the resulting data set with residue-level metadata, including the measure of energy we were interested in as well as physicochemical attributes, such as thermodynamic β-sheet and coil propensities^43^ for comparison.

### Counterfactual Sequence Generation and Structural Modeling

To test to what extent nanocdr-x predictions might be influenced by the residue position rather than the residue identity, we designed a counterfactual study using two representative nanobodies (PDB IDs: 7C8V_SR4 and 8ELO_C4). Artificial sequences were generated in two ways: (a) by shifting the native CDRs to three different, random unnatural positions along the sequence scaffold, and (b) by completely randomizing (scrambling) the entire sequence. The 3D structures of these artificial sequences were subsequently modeled using the AlphaFold3 Server,^14^ and their intramolecular interaction energy profiles were analyzed using the MLCE protocol.

## Results

### Deep Learning Model Performance

The architecture (Figure 1) trained on INDI^19^ achieved an accuracy higher than 0.995 already at 2 epochs, with minimal but steady increase until 4 epochs. No sign of overfitting can be reported, since the validation accuracy maintained values above the training accuracy until epoch 4 where the training was stopped (Supporting Information, 1.2).

**Figure 1:**
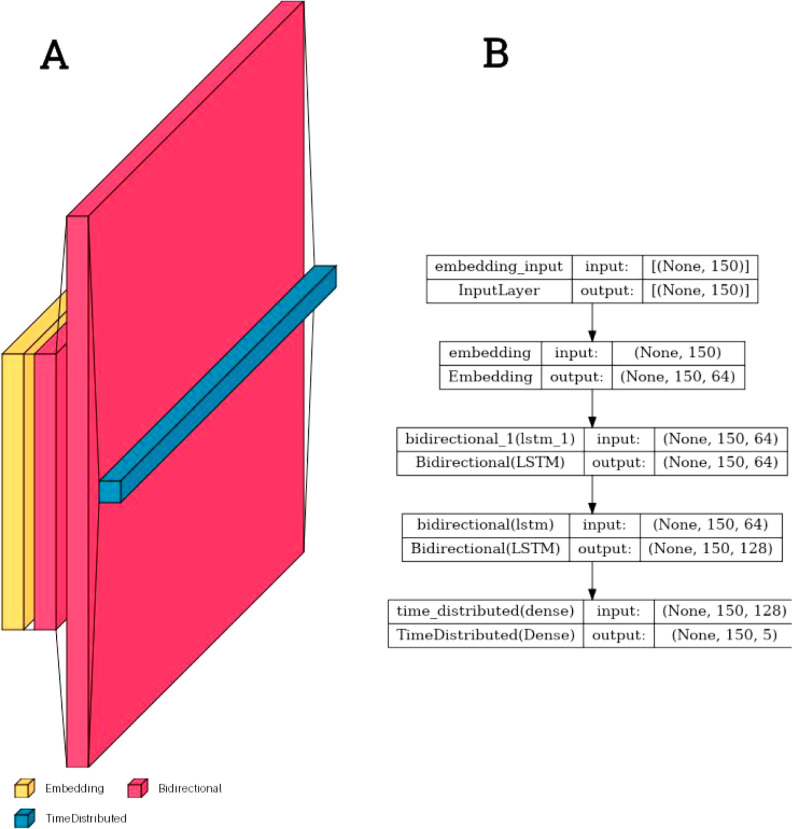
Nanocdr-x architecture. (A) Schematic 3D representation of the sequential architecture used for nanobody sequence labeling. The model is composed of an embedding layer (yellow), followed by two stacked bidirectional LSTM layers (red), and a time-distributed dense output layer (blue). This configuration allows the network to learn contextual information from both *N*- and *C*-terminal directions of the sequence. (B) Computational graph of the implemented architecture, showing the dimensionality and output shape at each layer. The embedding layer maps input tokens (sequence length 150) into a 64- dimensional dense space. The two BiLSTM layers, with 32 and 64 units respectively, process the sequence bidirectionally and return full sequences to preserve contextual dependencies. The final time-distributed dense layer applies a softmax activation to predict residue class probabilities across five categorical outputs (including padding).

The final validation accuracy reached 99.9% on the INDI data set. It is important to clarify that this metric represents the model’s ability to fit the training distribution and serves primarily as a diagnostic quality control to demonstrate the absence of severe overfitting. Naturally, predictive performance (evaluated in training accuracy vs validation accuracy) is expected to adjust when moving from the data set used to fit the model to strictly independent data (testing). The true predictive capability of the model on unseen, real-world sequences is therefore evaluated separately on an independent test data set, as detailed in the following section. The model thus trained was therefore saved for release along with the encoding and the decoding scripts, as well as interpretability tools, which we discuss later.

The software is distributed with a conda package easily accessible at *lescailab::nanocdr-x* and reference documentation has been made available at https://lescailab.github.io/nanocdr-x/where the source code can also be accessed.

A performance test at first installation showed that the analysis of 121 nanobodies (independent data set, see below) required 43 s of wall-clock time (real 0m43.061s) with a user CPU time of 5.696s.

### Independent Data Set Validation

We selected an independent data set of 121 nonredundant Nbs, with experimentally resolved structures to assess the model accuracy beyond the training and validation data, and to compare the predictions with consolidated methods in the literature., To ensure robustness and avoid biases, we cross-referenced our predictions with the Chothia,^9^ IMGT^11^ and AbM^10^ numbering schemes, evaluating the residue-level classification through confusion matrices. For this comparison, we encoded each sequence position into a class number: 0 for body (defined as any nanobody sequence position not classified as a CDR), 1 for CDR1, 2 for CDR2, and 3 for CDR3. Padding positions were excluded from the analysis.

A detailed residue-by-residue comparison against the Chothia annotation^9^ is summarized in the first confusion matrix shown in Table 1. The results show strong concordance between the predicted and reference loop regions, with most predictions falling precisely along the diagonal. Nanocdr-x exhibited near-perfect identification across all three CDRs, alongside highly accurate classification for non-CDR (body) sequences. Overall, this evaluation revealed that the model yields a global F1-accuracy score of 95.6%. Specifically, the model achieved a 99.8% agreement with IMGT definitions (Table 1, confusion matrix 2); this near-perfect match is expected and serves as a confirmation of the model’s learning accuracy, considering that nanocdr-x was trained on the INDI data set which utilizes IMGT numbering. Furthermore, the observed 94.8% agreement with AbM definitions (Table 1, confusion matrix 3) demonstrates that the model captures universal CDR features and is robust enough to generalize across different numbering conventions.

**Table 1:** Confusion Matrix Comparing Standard Numbering Schemes and Nanocdr-X Loop Encodings^a^

confusion Matrix 1						total expected (percentage predicted)
true label (Chothia)	class 0	11096	140	249	273	11758 (94.4%)
	class 1	0	835	0	0	835 (100%)
	class 2	0	0	695	0	695 (100%)
	class 3	2	0	0	1697	1699 (99.9%)
	total predicted	11098	975	944	1970	
	class 0	class 1	class 2	class 3		
	predicted Label (nanocdr-x)					
confusion matrix 2	total expected (percentage predicted)					
true label (IMGT)	class 0	10910	3	0	19	10932 (99.8%)
	class 1	0	956	0	0	956 (100%)
	class 2	2	0	936	0	938 (99.8%)
	class 3	0	0	0	1937	1937 (100%)
	total predicted	10912	959	936	1956	
	class 0	class 1	class 2	class 3		
	predicted label (nanocdr-x)					
confusion matrix 3	total expected (percentage predicted)					
true label (AbM)	class 0	10434	0	0	259	10693 (97.6%)
	class 1	237	959	0	0	1196 (80.2%)
	class 2	241	0	936	0	1177 (79.5%)
	class 3	2	0	0	1697	1699 (99.9%)
	total predicted	10914	959	936	1956	
	class 0	class 1	class 2	class 3		
	predicted label (nanocdr-x)					

a The table displays three separate confusion matrices evaluating nanocdr-x predictions against, respectively, Chothia, IMGT, and AbM annotations. Each cell reports the Number of residues assigned to a given class by the respective reference scheme (rows) and by nanocdr-x (columns). Values along the diagonals indicate concordant classifications, whereas off-diagonal values correspond to discrepancies.

Notably, this analysis excludes two specific nanobodies (PDB IDs: 7FBJ_17F6 and 7FBK_20G6) for which standard numbering tools are unable to provide a prediction, because they heavily rely on the alignment to databases, largely built on antibodies. It is crucial to highlight that, while traditional motif-based approaches failed to annotate these sequences, nanocdr-x successfully predicted their antigen-binding loops, we mapped the sequences identified as CDRs by nanocdr-x onto the crystallized structure available in the PDB, and they corresponded to the protein’s CDR regions. This underscores the capability of our deep learning approach to identify CDRs even in scenarios where standard numbering schemes fail, offering a significant advantage for the characterization of novel or noncanonical nanobodies.

### Benchmarking

To evaluate the performance of our tool (nanocdr-x), we sought to conduct a comparative analysis with the main tools described in the literature which mentioned CDR loop prediction in either antibodies or nanobodies. The comparison focused on approaches using deep-learning, with specific attention to those explicitly designed to handle nanobody sequences or to predict the CDR loops. The final selection of tools was guided by a systematic review of recent literature^7^ and includes: simpleDH3,^44^ AntiBERTa,^45^ AntiBERTy,^45^ ProtBERT,^46^ nanoBERT,^47^ ABlooper.^48^ However, our assessment revealed fundamental methodological differences that preclude a direct quantitative benchmark (e.g., comparing precision, recall, and F1-scores). These tools are designed for entirely different tasks and do not perform direct sequence-to-label CDR identification out-of-the-box. (a) Protein Language Models (PLMs) (e.g., nanoBERT, AntiBERTy, ProtBERT, AntiBERTa): these are pretrained models designed for masked language modeling or generating vector embeddings. They do not output CDR sequence classifications. Utilizing them for this task would require designing and training a novel classification head on top of their embeddings, which effectively constitutes building a new model rather than benchmarking an existing out-of-the-box tool. (b) ABlooper: this is a structural prediction tool primarily intended for paired antibody chains (Heavy and Light). It does not natively support single-chain modeling from raw, unnumbered sequences without relying on an external prenumbered input model (e.g., via ABodyBuilder2). Since nanocdr-x processes raw, unnumbered single-chain sequences, the inputs are inherently incompatible. (c) AlphaFold3 (AF3):^14^ as a structure prediction tool, AF3 outputs 3D atomic coordinates rather than sequence annotations. Extracting CDR sequences from an AF3-predicted structure strictly requires the application of a geometric or sequence-based numbering algorithm (such as ANARCI^24^). Therefore, a benchmark in this context would ultimately evaluate the accuracy of the numbering algorithm rather than the predictive capability of AF3 to identify CDRs as sequence labels. (d) SimpleDH3: this tool is an end-to-end deep learning model specifically designed to predict the 3D backbone conformation of the CDR-H3 loop. As a structural predictor, it outputs spatial coordinates rather than assigning CDR classes to residues directly from the primary sequence. To clarify these distinctions, we have systematically categorized these tools by their specific tasks, inputs, and outputs in Supporting Information, 5.1. In contrast to these approaches, nanocdr-x fills a unique niche: it was specifically designed to perform an end-to-end classification of nanobody sequences, assigning each residue to one of the four classes (body, CDR1, CDR2, CDR3) directly and solely from the primary sequence.

### Characterizing the Binding Loops

#### Structure-Energy Based Identification of CDRs (Paratope Prediction)

In parallel, we performed the prediction of CDRs on a database of 121 nonredundant Nbs known to bind the Receptor Binding Domain of Sars-CoV-2. As we reported in Materials and Methods, we chose Nbs on the basis of the availability of their experimental three-dimensional structure solved by either X-ray Crystallography or Electron Microscopy and deposited in the Protein Data Bank. After structure preparation (see Materials and Methods), we predicted paratope regions on each Nb structure in isolation using our in-house method MLCE (Matrix of Low Coupling Energy^30,32,36,38,40,42^ ). Our working hypothesis is that CDRs need to have a peculiar energetic signature, allowing them to adapt to the binding partner via significant conformational changes and sampling of diverse structural populations, from which the binding states can be selected. This type of behavior identifies CDRs as fuzzy regions. Therefore, it is reasonable to hypothesize that their residues are weakly coupled with the rest of the protein from the energetic point of view. The formation of a localized network of weak interactions is in fact a distinctive trait of fuzzy substructures. As a consequence, to identify them, we used an energy-pair decomposition approach, namely MLCE.

MLCE analyzes the interaction energies between all amino acid pairs within a protein with a solved 3D structure. First, an MM/GBSA calculation is performed to compute the nonbonded part of the potential, i.e. electrostatic and van der Waals interactions and solvent effects, yielding an *N* × *N* symmetric interaction matrix *M*_*ij*_ for a protein of *N* residues. The matrix is then diagonalized and reconstructed through eigenvalue decomposition to identify regions of strong and weak energetic coupling (see Figure 2). Since it can be assumed that the first (most negative) eigenvalue and the associated eigenvector contain most of the information on the stabilizing interactions of the protein (see Materials and Methods), we then analyzed the contribution of each residue to the most negative eigenvector. As an example, values calculated for Nb1A7 from PDB structure 7FAT are reported on the *y* axis (energetic contribution) in the plot in Figure 3A, while residue numbers are listed on the *x* axis. Highest energy values correspond to a high contribution to the most negative eigenvector and are characteristic of stabilizing regions of Nbs (i.e., the folding core or stability core), while lowest values are distinctive of weakly coupled regions, normally associated with PPI surfaces. We consistently identified regions with low values by setting a threshold for each Nb calculated as $\frac{1}{\sqrt{N}}$ . This limit represents an ideal situation in which each of the *N* residues contributes equally to the first eigenvector (flat eigenvector hypothesis). Consequently, residues with higher energy values compared to the threshold are important for the stabilization of the protein fold and their energetic contribution is represented as blue bars in Figure 3A, while residues with lower energy values belong to regions with more conformational variability, and thus possibly important for the interaction with partners. CDR1/2/3 have the lowest values in the plot and their corresponding bars are depicted in red. The contribution of each residue to the first eigenvector can be projected on Nb1A7 structure, as shown in Figure 3A–E: the color spans from blue (stabilizing regions) to red (weakly coupled regions). This trend, consistently observed over the 121 Nbs we analyzed, indicates that CDR regions always correspond to residues weakly coupled to the rest of the protein. This can be observed in another example, reported in Supporting Information, 2.1, where energetic contributions values are plotted for Nb Sb23 from PDB structure 7A29. Along with the CDRs, other regions weakly coupled to the rest of the structures are the *C*- and *N*- terminal loops, which are decoupled by their very nature from the rest of the structure, and often the loop located between residues 74 and 78, whose role is under debate to such an extent that it has been recently defined as “the fourth CDR”.^49^

**Figure 2:**
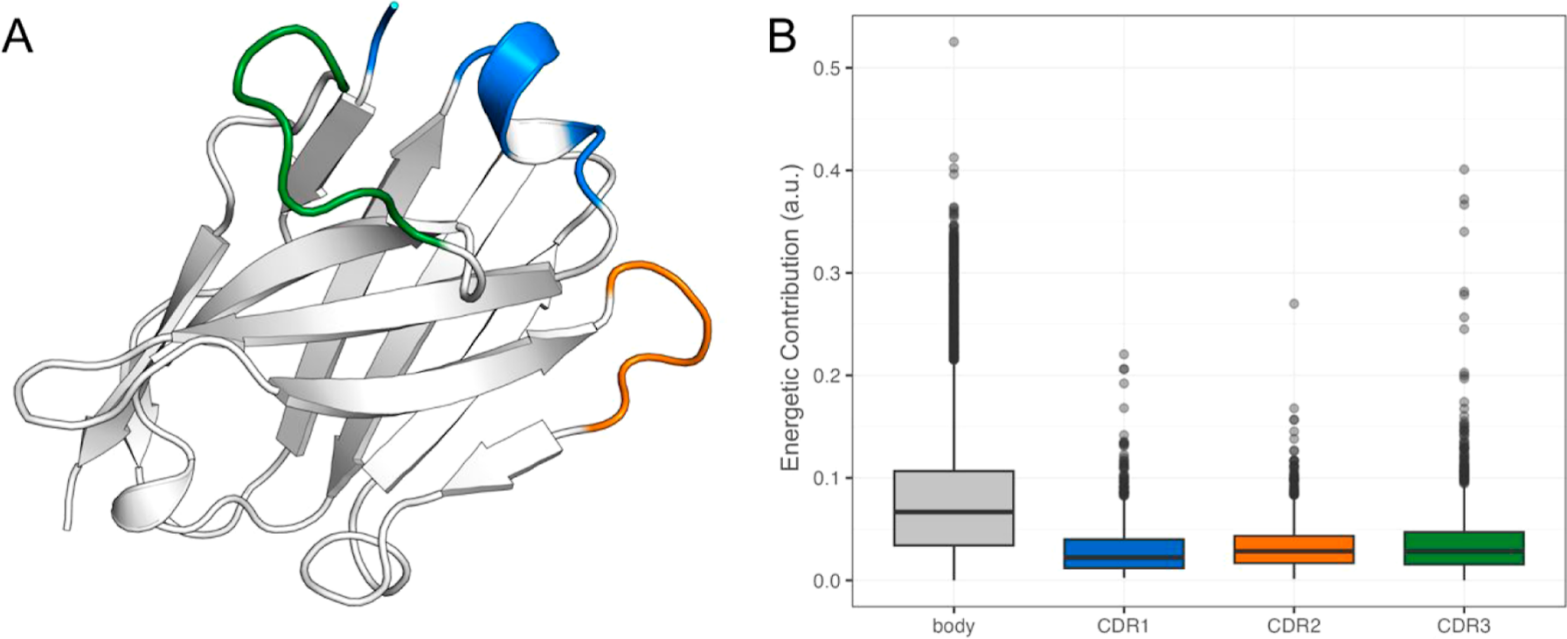
Structure- and energy-based identification of CDRs. (A) Predicted interaction regions using MLCE on the structure of nanobody 7KKK_Nb6 (PDB ID: 7KKK). The protein fold is shown in cartoon representation (gray), with predicted interacting patches in orange, blue, and green. These respectively correspond to CDR1, CDR2, and CDR3, showing that MLCE correctly identified CDRs as weakly coupled, flexible regions. (B) Distribution of interaction energy values for residues classified as body or CDRs. Boxplots show significantly lower energy values in CDR1–3 compared to body residues (ANOVA, *F*(3, 14,983) = 682.6, *p* < 2.2 × 10^–16^), indicating that CDRs consistently display a characteristic energetic signature of weak coupling.

**Figure 3:**
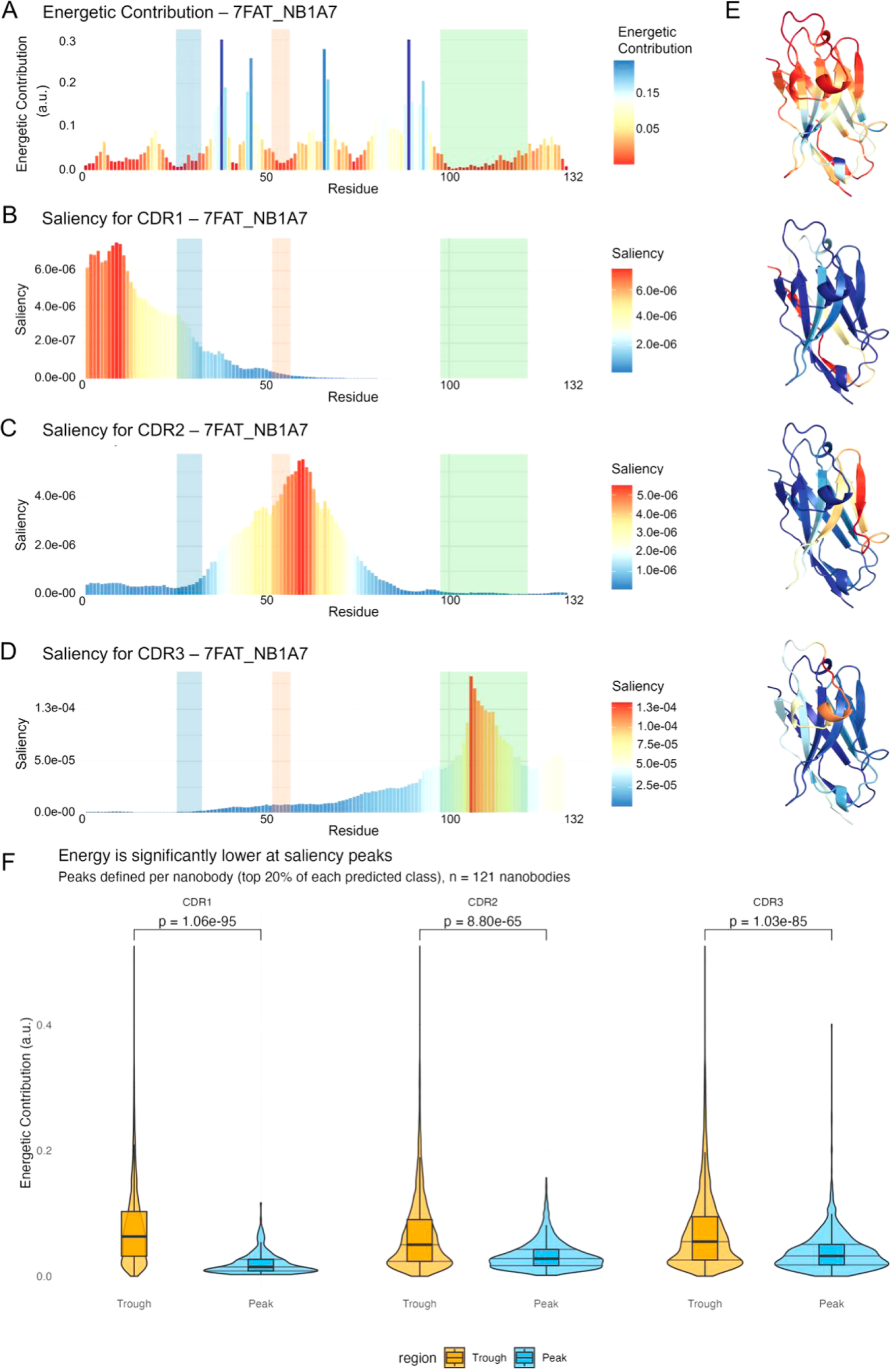
Saliency mapping of nanobody 7FAT_NB1A7 (PDB: 7FAT) in relation to structural energy. Residue-level saliency­(3B,3C,3D) scores, derived from model gradients, indicate the importance of each residue for each CDR classification. Regions with high saliency overlap with low-energy­(3A) residues of the structure and are concentrated within the CDR loops. This alignment highlights the model’s ability to focus on structurally flexible yet functionally relevant areas. Compared to state-of-the-art methods, the approach achieves improved localization of CDRs, with predictions supported by structural stability mapping. The right panel (3E) shows the projection of values from the respective plots (3A, 3B, 3C, 3D) on the 3D structure of 7FAT_NB1A7. In panels 3A, 3B, 3C, 3D, canonical CDRs annotated with Chothia are highlighted as blue, red, and green rectangles, respectively. The violin plot in Figure 3F illustrates the joint analysis of saliency scores and energetic contributions across the data set. Sequence positions with high predictive saliency (peaks) show strictly lower coupling energy values compared to trough regions, with the difference being highly significant across all CDRs (Wilcoxon rank-sum test, *p* < 10–64).

In the last step of the MLCE procedure, the interaction matrix *M*_*ij*_ is filtered by the contact matrix *C*_*ij*_ (*see* Materials and Methods) in which potential interacting patches are rigorously identified as sets of close-by residues weakly coupled with the rest of the protein. These characteristics are always typical of CDRs, as they are important interacting regions, and can also be considered as hallmarks of fuzziness. In this context, we envision that calculations like the ones presented here could aptly be used to predict CDRs from simple structural information.

Importantly, patches identified on Nbs often include residues belonging to CDRs according to Nanocdr-x predictions (*see* previous paragraphs). In Supporting Information, 3.1, the results of our MLCE-guided paratope prediction on Nbs show that in 112 out of 121 Nbs the predicted patches include one or more CDR3 residues (90,91% success). As an example, in Figure 2A interacting regions predicted using MLCE are highlighted on the structure of 7KKK_Nb6: as it can be observed, CDR loops are correctly included in the patches. In Figure 2B, a boxplot reports the distribution of interaction energy values across body residues and the three CDRs. A one-way ANOVA confirmed that the differences among residue classes are highly significant (*F*(3, 14,983) = 682.6, *p* < 2.2 × 10^–16^), supporting the hypothesis that CDR loops correspond to weakly coupled, energetically uncoupled regions when compared to the structural core of nanobodies.

To provide a further structural validation independent of the results observed on the 121 Nbs experimental data set, we selected 7 new sequences representative of highly divergent clusters obtained from the INDI data set. We modeled their 3D structures using the AlphaFold3 Server^14^ and subsequently calculated their energy profiles using MLCE. As reported in Supporting Information, 3.2, this analysis confirms that CDR residues are consistently identified as energetically decoupled from the rest of the Nb scaffold, even across highly diverse sequences.

### Explainability

The excellent performance of our model prediction addresses one of the main challenges we sought to resolve, i.e. making CDRs predictions accessible and quick. However, this accuracy solely based on sequence context opened an additional opportunity to better understand the molecular and biochemical characteristics of those regions. The structural characterization of CDRs obtained using the energy decomposition method presented in the previous paragraph revealed the fuzzy nature of these regions. We therefore analyzed the performances of our model in terms of saliency and hidden states to capture the internal representation of Nbs and the relationship with structure-based energetic calculations.

### Saliency

First, we focused on the relationship between saliency and energy measures. Saliency is computed (see Materials and Methods) on the model gradient, to assess the average importance of each residue in allowing the model to predict each class. Figure 3A shows the values of the energy measured along nanobody NB1A7 from PDB structure 7FAT; Figure 3B plots the importance of different residues in the prediction of CDR 1 positions; Figure 3C plots the importance of each residue in achieving the prediction of the CDR2, and Figure 3D plots the average importance of residues in allowing the model to identify CDR3. The three plots combined show that the most important positions for the neural network to identify the binding loops correspond to areas of low energy, and only in part to framework positions. A central element of the saliency analysis is that the highest importance in predicting each loop seems to reside in the loop itself, rather than in surrounding regions. This is significantly different from the established methods in the literature, which cannot identify randomly generated sequences such as the binding loops, and therefore focus on identifying loose patterns in the surrounding regions. Figure 3E provides a structure-based context to these observations. The plot shows the importance of the loops themselves in predicting the binding regions, together with flanking sequences which, according to their energy measures, might contribute to the stability of the molecule. Another example is reported in Supporting Information, 2.1, where saliency values and energetic contributions are plotted for Nb Sb23 from PDB structure 7A29.

To quantitatively validate the relationship between sequence saliency scores and energetic contributions across our data set, we conducted a comprehensive joint analysis. Sequence positions were classified into “peak” and “trough” regions. Specifically, peaks were defined as residues falling in the top 20% of the saliency distribution (calculated per nanobody and per CDR prediction) that match the predicted CDR class. Conversely, troughs were defined as residues in the lower 80% of the saliency distribution falling outside the target prediction. A one-sided Wilcoxon rank-sum test confirmed a highly significant difference across all three CDRs (*p* < 10^–64^), demonstrating that sequence positions with high predictive saliency (peaks) are consistently characterized by strictly lower coupling energy values compared to trough regions. This global trend is illustrated in Figure 3F.

### Hidden States Analysis

To further understand if the molecule flexibility was indeed what the architecture has captured, we decided to analyze the internal representation of the nanobody, learned by the model. This is done by extracting the numerical representations (hidden states) of each residue built by the last layer of the network, before the classification output. Given the dimensionality of the last LSTM layer, each amino acid is represented by a vector of 128 values. Therefore, dimensionality reduction was used to facilitate the interpretability and visualization of such representations and assess any relationship with the energy measure as well as with other residue characteristics annotated from the literature.

We performed a principal component analysis (PCA) of the hidden states thus extracted, evaluating the distribution of each nanobody position along the first two principal components. The PCA plot shows a clean separation of the different CDRs on the PCs plane, thus confirming the ability of the model to separate (i.e., predict) the different classes.

We then added the energy measure as a third dimension, to better assess the relationship not only with the prediction (saliency) but also with the model representation of the nanobody (hidden states). Figure 4A shows that the denser areas of the residues predicted as the three CDRs correspond to lower energy regions. Alternatively, adding the thermodynamic beta-sheet propensity^43^ as a third dimension (Figure 4B) shows a randomly scattered distribution of the very same residue representations. The comparison of the two annotations shows that the classical thermodynamic structure propensity measures do not cluster with the CDRs representations, while most clearly the fuzziness of the molecule does.

**Figure 4:**
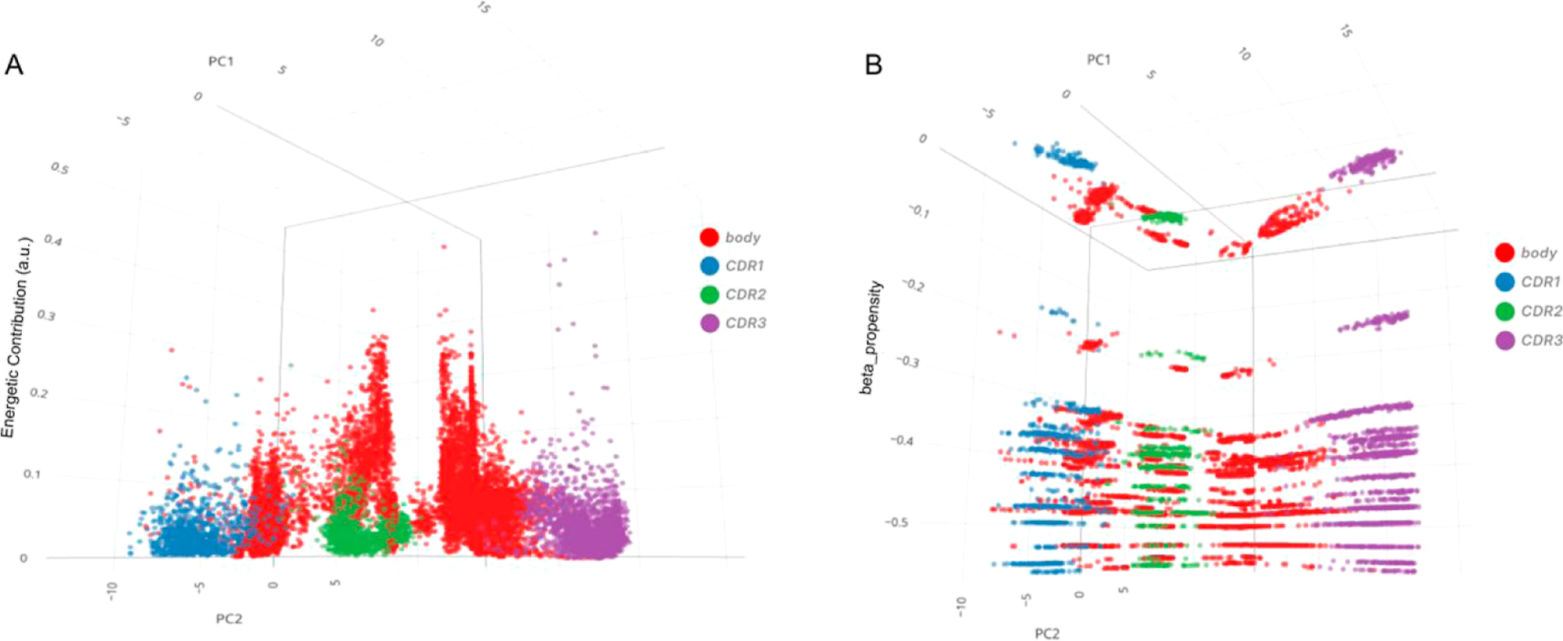
Projection of hidden state representations onto structural properties. Hidden states from the neural network capture how the model represents nanobody sequences. When projected onto β-sheet propensity values (B), the embeddings appear evenly scattered and do not separate CDRs from framework regions. In contrast, projection onto structural energy decomposition (A) results in distinct clustering that aligns with CDR classification. This suggests that the features the model learnt align with the intrinsic “fuzziness” of nanobody regions, as reflected by their energy scores, thereby supporting accurate loop identification.

### Counterfactual Analysis of Positional Bias and Structural Fuzziness

The absolute position of each residue in the nanobody is not encoded in the input tensor of our model, and LSTM layers are not designed to learn the position as such, but rather the context.^50^ However, to directly address whether the model genuinely learnt residue properties, and in particular structural “fuzziness”, rather than learning absolute positional biases (e.g., standard sequence anchors), we evaluated nanocdr-x predictions on artificial sequences generated by altering the nanobodies 7C8 V and 8ELO_C4 (CDRs shift or sequence randomization, see Materials and Methods, and Supporting Information, 4.1). If the model simply relied on a positional information or a coordinate system, it would predict CDRs in a canonical absolute position regardless of the sequence modifications introduced. Interestingly instead, the model identified entirely new sequence stretches as putative CDRs in the nanobodies where the original sequences had been shifted: the predicted CDRs are located in similar topological areas but show different lengths and completely different flanking amino acids (see Supporting Information, 4.2) when compared to the original ones. The fact that the lengths, sequence as well as boundaries of these predicted CDRs vary, despite the absence of the original start/end sequence patterns, strongly indicates that the model has not learnt the anchor motifs patterns nor the absolute CDR positions, and confirms what is known from LSTM literature, i.e. this architecture cannot rely on such information to yield its predictions. This is further confirmed by the results of the analysis on the completely randomized sequences, where nanocdr-x appears unable to produce coherent and continuous CDR predictions (Supporting Information, 4.2). To take this a step further and test the biophysical relevance of these predictions, we modeled the 3D structures of these artificial (i.e., shifted or randomized) sequences using the AlphaFold3 Server.^14^ We then calculated their energy profiles with MLCE (see Supporting Information, 4.3). As expected, they folded into highly disordered structures with numerous unnatural loops, prompting MLCE to identify multiple distinct uncoupled, low-energy regions. Crucially, when mapping the sequence-based nanocdr-x predictions onto these 3D structures, the network, with only one minor exception, consistently targeted regions that structurally fold into loops (Figure 5, A and B). Even with the MLCE values suggesting the presence of more low-energy residues compared to what would be found in real nanobodies, the residues classified by nanocdr-x as CDRs show lower values compared to rest of the protein (Figure 5C—*t*-test *p*-value 2.792 × 10^–12^) This finding demonstrates that even when the native nanobody scaffold is disrupted and the canonical sequence patterns are lost, the neural network still recognizes regions characterized by flexibility and low energetic contribution.

**Figure 5:**
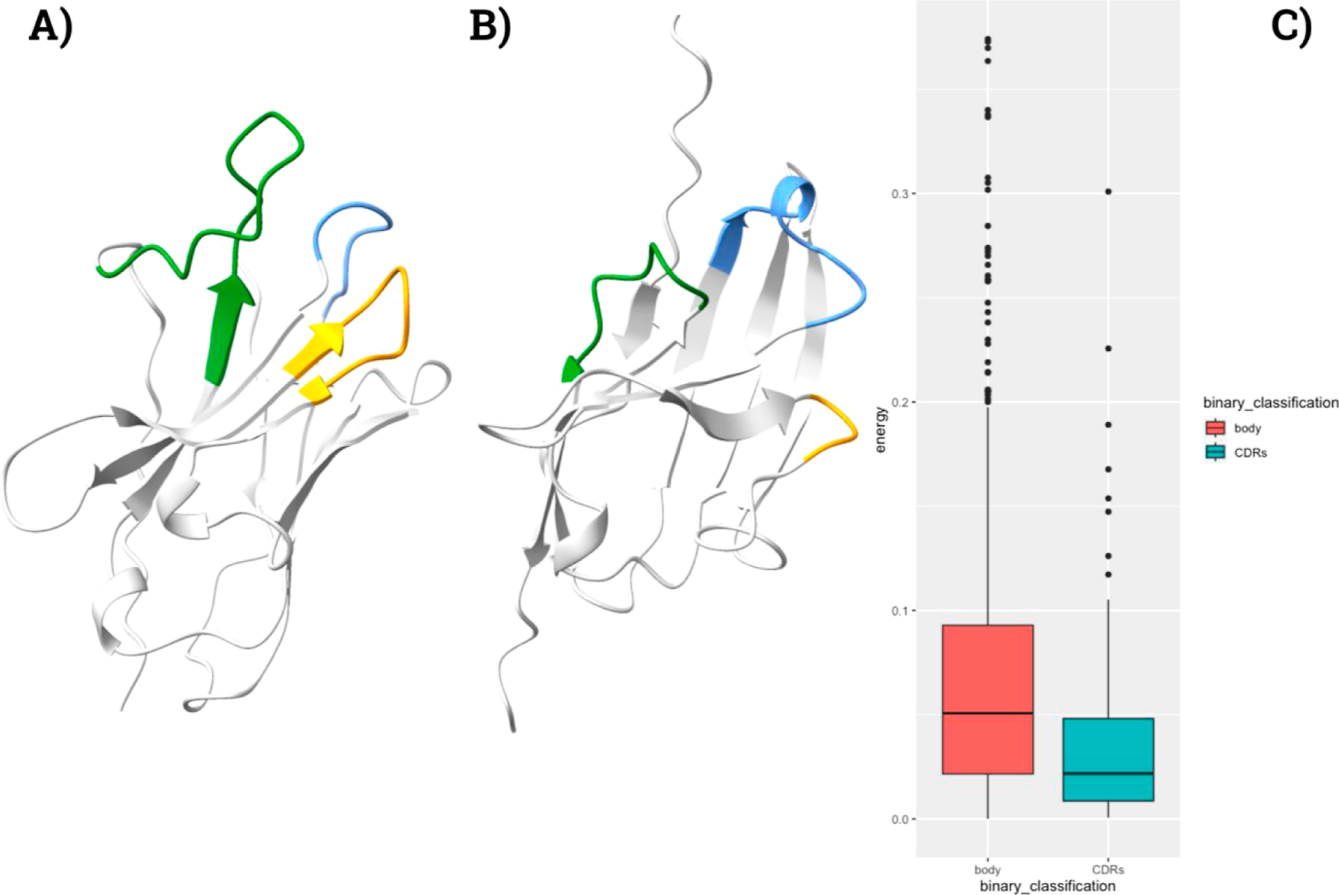
Counterfactual analyses of nanocdr-x predictions. The figure reports the results of nanocdr-x predictions on artificial versions of 7C8 V and 8ELO_C4 nanobody sequences, obtained by shifting the original CDR sequences along the nanobody. The resulting 3D structure has been predicted with AlphaFold3, and MLCE has been used to calculate the residues coupling energies of these structures. Panel (A) shows the location of CDRs predicted on the modified sequence of 7C8 V. Panel (B) shows the location of CDRs predicted on the modified sequence of 8ELO_C4 (CDR1: green, CDR2: blue, CDR3: orange). The boxplot in panel (C) compares the distribution of coupling energies of residues predicted to be in CDRs or outside (body) among all versions generated by CDR shift of the two nanobodies (3 each): the difference is significant with a *t*-test p-value of 2.792 × 10^–12^.

## Discussion

Nanobodies (Nbs) have emerged as an interesting class of antigen-binding biomolecules, with applications that span from next-generation therapeutics to precision diagnostics. Nbs are relatively small single chain molecules, which makes them versatile tools easier to produce and handle compared to larger multichain antibodies.

Understanding the determinants of their evolution and of selective antigen binding is crucial to rationally engineer novel nanobodies. In this context, investigations have long concentrated on clarifying antigen-binding mechanisms through structure-based methods, which rely on molecular docking, homology modeling, and most recently on the use of AI-driven approaches, such as AlphaFold2 and AlphaFold3.^13^

A first critical step toward uncovering the key determinants of Nbs mechanisms, and possibly developing rules for their design, resides in the unbiased identification of Complementarity-Determining Regions (CDRs), i.e. those loops responsible for the interaction with the antigen. The key question is whether a unifying framework can be developed integrating sequence and structural properties to capture the emergence of the unique functional features that characterize CDRs residues.

Yet, characterizing CDRs, especially CDR3, remains a key challenge: their sequences arise from an inherently random mechanism resulting from the *V­(D)­J* recombination, as well as from further somatic mutations arising during the affinity maturation process. The structure of these loops tends to be flexible and disordered, and predicting their localization may turn out to be challenging even for state-of-the-art methods looking at the 3D context such as AF3.

In this work, we set out to define a general and unbiased framework for CDR identification, simultaneously addressing both sequence and structure. The aim is to define a general signature that connects such properties while explaining the intrinsic specificity of CDR regions in antigen recognition.

From a sequence perspective, we worked on the hypothesis that using predefined schemes for CDRs identification was in (strong) contrast with the intrinsic variability of CDRs sequences, and in particular of CDR3. We developed nanocdr-x, a deep learning architecture trained on more than 11 million nanobody sequences. For the first time, a model could robustly predict CDRs sequences and their localization, with high accuracy. This approach fills a methodological gap: nanocdr-x is the only available tool that provides an end-to-end, open source, and easily accessible solution for large-scale automatic CDRs identification directly and solely from sequence data.

Crucially, our evaluation highlighted that predicting CDRs based on fixed sequence motifs is intrinsically tied to the chosen nomenclature. Initial validations against the Chothia scheme showed minor boundary discrepancies; however, cross-referencing our predictions with IMGT and AbM definitions revealed that these mismatches are largely artifacts of conflicting labeling schemes rather than shortcomings of the neural network. Indeed, when we evaluated our predictions against the IMGT definitions, which align with the model’s training data, nanocdr-x achieved near-perfect boundary recognition, further demonstrating its robust learning of loop architectures. Furthermore, this comparison exposes the primary limitation of relying on traditional Multiple Sequence Alignments (MSAs) and Hidden Markov Models: their strict dependence on database homology. If a novel nanobody exhibits highly divergent or unusually long CDR3 loops, classical alignment-based predictions fail. This occurred precisely with two experimentally resolved nanobodies in our structural test set (PDB IDs: 7FBJ_17F6 and 7FBK_20G6): standard MSA-based tools were unable to process them, whereas our single-sequence deep learning approach successfully identified their antigen-binding loops. This highlights the concrete methodological advantage of our model over traditional alignment-based tools, demonstrating its capability to generalize beyond simple homology.

Importantly, this result focused our attention on the molecular properties of nanobodies and what the model had learnt. Nanocdr-x does not need any additional information, such as structure or residue annotations. The saliency analysis, i.e. measuring the importance of each residue for the model to achieve its prediction, placed the highest saliency values within the CDRs themselves. This could only be interpreted with the existence of specific properties which are intrinsic to the CDR residues, and which the neural network could uniquely recognize to achieve the successful prediction of the CDR sequence and localization in the protein.

We next asked whether sequence features may propagate into structural properties characteristic of CDRs. CDRs are known to sample diverse conformational ensembles, from which a preferred state is selected upon antigen binding. Such a conformational selection mechanism parallels the behavior of intrinsically disordered regions, enabling efficient recognition and adaptability to the target. We reasoned that this flexibility arises when residues within CDRs are only weakly coupled to the Nb global fold, allowing them to adopt multiple conformations and accommodate high mutational variability without compromising the protein’s structural integrity. Antigen engagement would then stabilize otherwise dynamic residues, favoring the formation of a functional complex.

To identify CDRs from a structural standpoint, we applied the Matrix of Low Coupling Energy (MLCE) method,^34^ which analyses intramolecular interaction energies across all residues of a protein with an available structure. MLCE assumes that interacting patches are defined by suboptimal intramolecular energetic pair-interactions, conferring flexibility and adaptability to binding partners. Using only the 3D structure of isolated nanobodies as input, MLCE consistently mapped CDRs as weakly coupled regions, without requiring prior knowledge of sequence motifs. These loops emerged as energetically decoupled from the protein core, supporting the view that low coupling underpins their conformational plasticity, capability to support high mutation rates, and functional adaptability.

Strikingly, our two completely independent methodologies pointed to the same conclusion, prompting us to investigate how they could be interpreted in light of each other. The explainability analysis of the deep-learning model clearly showed that the intrinsic properties recognized by the neural network in CDRs aligned closely with the low-energy values identified by MLCE. Unsurprisingly, when testing nanocdr-x explanations with conventional residue properties such as beta-sheet propensity, they were not able to account for the predictions achieved by the model.

These findings strengthen the concept of fuzziness as a defining characteristic of CDR regions in nanobodies. The idea that flexibility and lack of thermodynamics consensus characterize complementary determining regions is an increasingly consolidated concept in structural immunology.^18^ This paradigm was strongly confirmed during the SARS-CoV-2 pandemic, where paratope fuzziness proved crucial for developing efficiently neutralizing antibodies against diverse variants.^51^

In this context, an emerging area of literature is increasingly focused on intrinsically disordered regions (IDRs) involved in protein–protein interactions.^15^ These particular substructures are disordered in isolation and select optimal configurations upon binding to their partner. This feature has been shown to play a key role in recognition processes across different scales.^52^ A relevant example in this context is represented by the formation of the chemokine heterodimer between CXCL12 and frHMGB1.^16^ The realization that fuzzy regions are widespread in protein mechanisms also recently attracted attention for drug development.^53^ The interpretability of nanocdr-x representations consolidates this view, showing that this is likely such an intrinsic feature of CDR residues that a neural network can learn it just by analyzing the primary sequence of these proteins.

Based on our results therefore, we propose a model where CDRs are inherently fuzzy: in this picture, fuzziness is a key feature in determining Nbs polyreactivity, since it can efficiently facilitate adaptation to different antigens. Since CDRs rigidify upon affinity maturation^54^ paratope fuzziness may be the decisive feature determining which Nbs (and potentially antibodies) progress to this stage, before rigidity is imposed to enhance the affinity for a specific epitope, via the acquisition of mutations that energetically stabilize the complex.

Fuzziness is not noise, it is function. Chaos here is adaptive. Disordered regions are all but random: their fuzziness is indeed the key property which confers the necessary adaptability to be selected as the most effective binding protein to an antigen target.

Assessing the “fuzziness” and conformational dynamics of CDRs has recently become the direct focus of other advanced structure-based deep-learning tools.^55^ Our work complements these advancements by offering novel sequence-to-label deep learning evidence, which strengthens such recent understanding of protein structures and protein binding regions. Beyond this conceptual advance, it paves the way for rational in-silico design strategies for antibodies and nanobodies.

## Limitations

The work we present is open to further improvements, by addressing a few of the current limitations. The INDI data set used for training exhibits a species bias with a predominance of sequences derived from *Lama glama* and *Vicugna pacos*, reflecting the common use of these animals in nanobody discovery campaigns. While the data set offers a wide variety of loop lengths and residue diversity, which is more critical than a balanced species distribution, the inclusion of additional species in the future might further improve our tool. Additionally, the INDI database contains highly redundant sequences, particularly originating from NGS data sets. The current performance of nanocdr-x measured on experimentally resolved structures showed that, despite these potentially limiting factors, the model generalizes effectively and is ready to be deployed. Furthermore, our explainability framework has not tested a correlation of saliency and hidden states with all potential intrinsic features of nanobody residues. While our findings point to the recognition of structural fuzziness as the primary driver of the model’s predictions, the network might also capture additional, yet-undefined sequence or structural features in addition to low interaction energy.

Finally, we should also highlight that MLCE does not currently handle multichain complexes: this does not affect the current analysis because nanobodies are single chain, but addressing this limitation in the method will allow us to expand the present study to the general realm of Antibodies in the future.

## Conclusions

In conclusion, we present an integrated sequence-structure framework for the unbiased identification and interpretation of nanobody CDRs. Using nanocdr-x, a deep learning model trained on more than 11 million sequences, we demonstrate that CDR regions can be accurately predicted directly from sequence without relying on predefined numbering schemes or homology-dependent MSAs. Independent structural analysis using pair-residue energy decomposition, as implemented in MLCE, reveals that these same regions correspond to energetically weakly coupled segments of the nanobody fold, pointing to their intrinsic conformational plasticity and adaptability to emerging partners. The convergence of these orthogonal approaches indicates that CDRs are characterized by an underlying thermodynamic signature of structural “fuzziness,” which enables mutational tolerance, conformational adaptability, and encodes for effective antigen recognition. Together, these findings suggest that fuzziness constitutes a general principle of nanobody paratope function and provide a conceptual and computational basis for future antibody and nanobody analysis, characterization and eventually design strategies.

## Supplementary Material



## Data Availability

The data used to develop the deep learning model are available on INDI,^19^ and the data used to carry out the analysis are available on PDB, with the identifiers mentioned in Supporting Information, 3.1. The code is openly accessible and documented on GitHub at https://lescailab.github.io/nanocdr-x/­(Zenodo DOI 10.5281/zenodo.17639531) and available as a conda package at lescailab::nanocdr-x. The code for MLCE is openly accessible and documented on GitHub at https://github.com/colombolab/MLCE (Zenodo DOI 10.5281/zenodo.17640724). The code used to perform the independent data set validation and to create the matrix table is openly accessible and documented on GitHub at https://github.com/lescailab/nanocdrx-validation.git.

## Notes

Nanocdr-x model training Nanocdr-x explainability analysis Prediction results: details Counterfactual analyses: modified sequences Benchmarking (PDF)
