# Supplementary material for: Adaptive Disorder as the Hallmark of Nanobodies Antigen-Binding Loops
Source: J Chem Inf Model. 2026 May 22;66(11):6644–58. doi: 10.1021/acs.jcim.6c00716 (PMC13250985; doi:10.1021/acs.jcim.6c00716)
Supplement: Supplementary file 1 [file ci6c00716_si_001.pdf]

Supplementary Information for

Adaptive disorder as the hallmark of nanobodies antigen-binding loops

Davide Bagordo[1,*], Gauthier Trèves[2,*], Mariangela Santorsola[1], Giorgio Colombo[2,+], Francesco Lescai[1,+]
[1]Department of Biology and Biotechnology "L. Spallanzani", University of Pavia, Pavia, Italy
[2]Department of Chemistry, University of Pavia, Pavia, Italy

[*]*these authors have contributed equally to the work*
[+]correspondence should be addressed to:
francesco.lescai@unipv.it
g.colombo@unipv.it

Table of Contents

1. Nanocdr-x model training *2*

1.1 Training parameters 2

1.2 Training and validation performance of the nanocdr-x model across epochs 2

2. Nanocdr-x Explainability Analysis *3*

2.1 Saliency mapping of nanobody 7A29_Sb-23 (PDB: 7A29) in relation to structural energy. 3

2.2 UMAP of Hidden States colored by Interaction Energy 4

3. Prediction Results: details *5*

3.1 Predicted interacting residues and CDR assignments for the 121 nanobody structures analyzed 5

3.2 Predictions on Independent Dataset 9

4. Counterfactual Analyses: modified sequences *10*

4.1 Shifted and randomised 7C8V_SR4 and 8ELO_C4 nanobody's sequences 10

4.2 Shifted and randomized nanobody sequences from 7C8V_SR4 and 8ELO_C4 predicted by NanoCDR-X 11

4.3 Shifted and randomized nanobody sequences from 7C8V_SR4 and 8ELO_C4 predicted by the MLCE. 12

5. Benchmarking *14*

5.1 Comparisons with other deep-learning approaches 14

1. Nanocdr-x model training

1.1 Training parameters

The model was trained using the Adam optimiser with a learning rate of $1*10^{-4}$ and a clip value of 1.0 to prevent gradient explosion. We employed sparse categorical crossentropy as the loss function. Regarding class imbalance handling, we did not apply differential weights between "Body" and "CDR" classes, as the model demonstrated robust convergence without forcing class-specific penalties. However, we implemented a strict masking strategy within the training loop to handle variable sequence lengths: a weight of 0 was assigned to padding tokens (PAD) and a weight of 1 to real residues.

1.2 Training and validation performance of the nanocdr-x model across epochs

(A) Training and validation loss over four epochs. Both curves converge rapidly, with minimal loss reduction after epoch 2.
(B) Training and validation accuracy across epochs. Validation accuracy remains consistently higher than training accuracy, indicating the absence of overfitting. The model achieved a final validation accuracy of 99.9% on the INDI dataset and was stopped after 4 epochs.

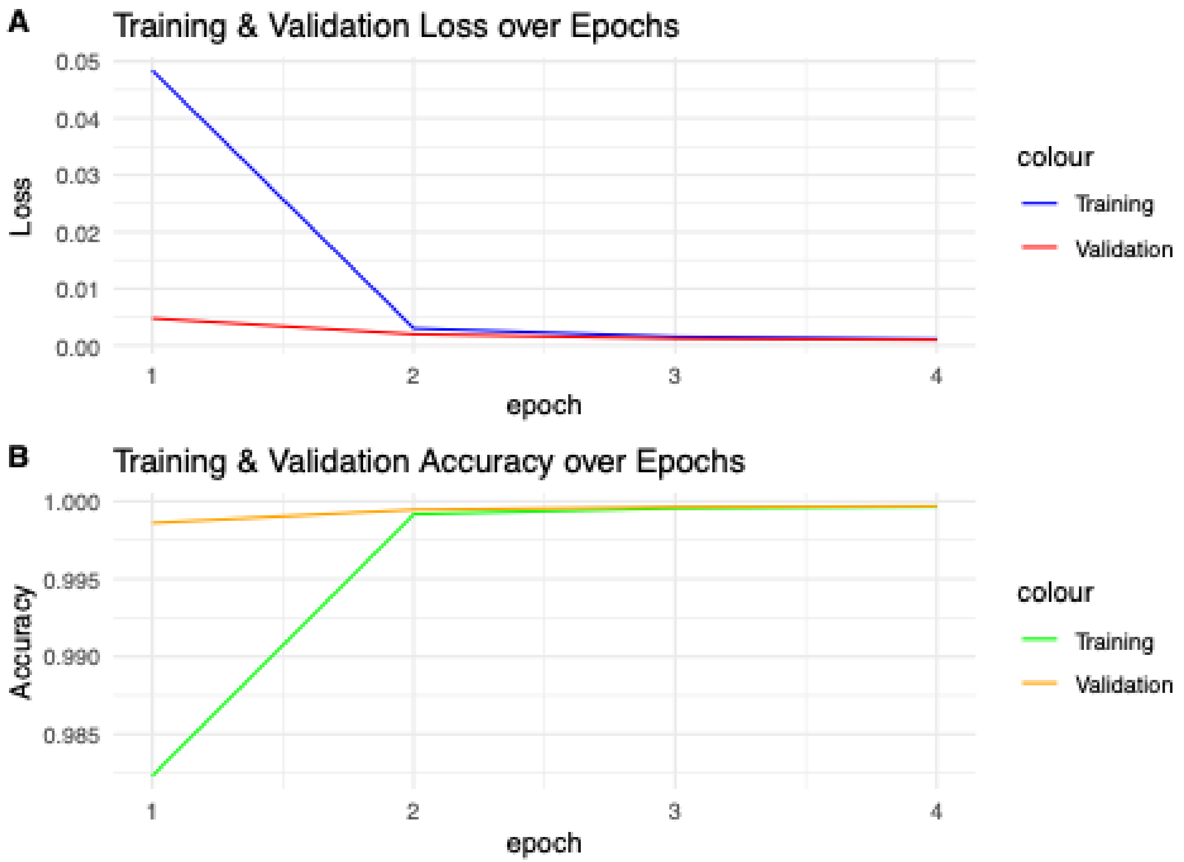

2. Nanocdr-x Explainability Analysis

2.1 Saliency mapping of nanobody 7A29_Sb-23 (PDB: 7A29) in relation to structural energy.

Residue-level saliency(2B,2C,2D) scores, derived from model gradients, indicate the importance of each residue for CDR classification. Regions with high saliency overlap with low energy(2A) residues of the structure and are concentrated within the CDR loops. This alignment highlights the model's ability to focus on structurally flexible yet functionally relevant areas. Compared to state-of-the-art methods, the approach achieves improved localisation of CDRs, with predictions supported by structural stability mapping. The right panel (2E) shows the projection of values from the respective plots (2A, 2B, 2C, 2D) on the 3D structure of 7A29_Sb-23.

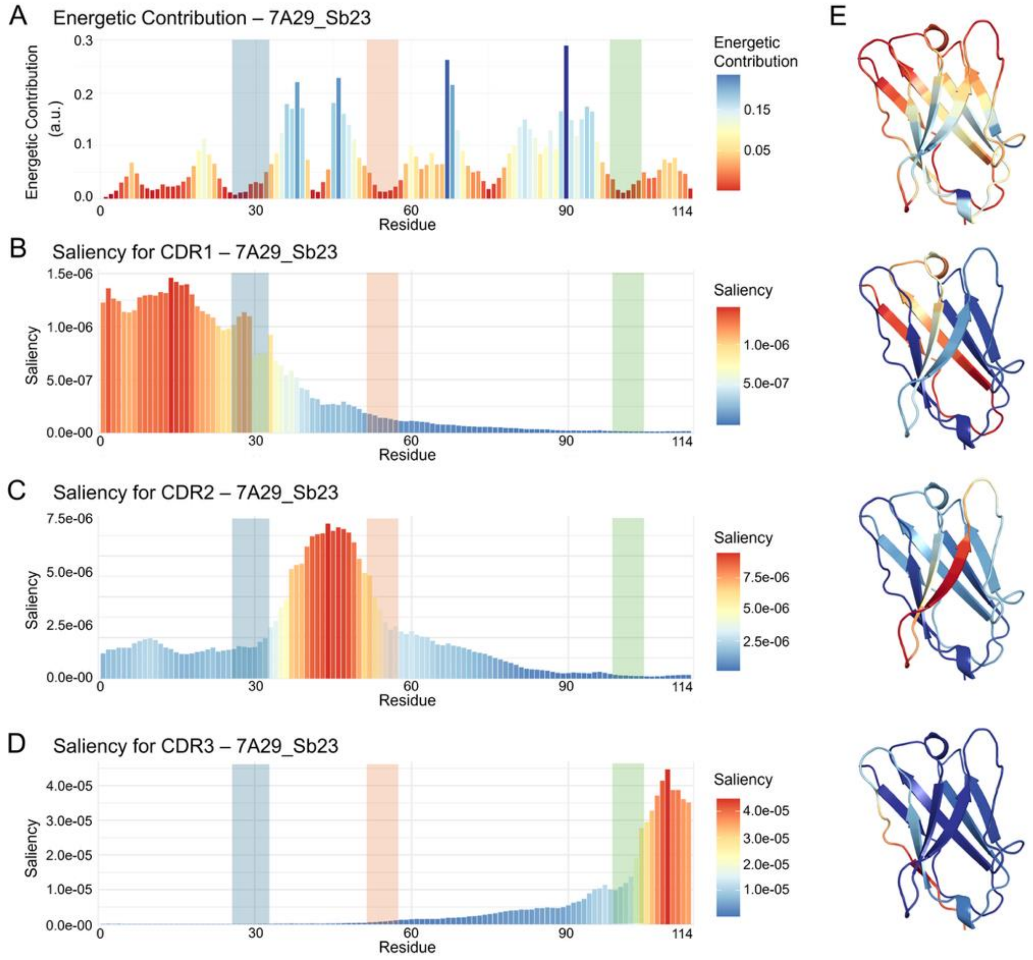

2.2 UMAP of Hidden States colored by Interaction Energy

Hidden states representations extracted from the second BiLSTM layer have been analysed with a dimensionality reduction approach meant to preserve local relationships (UMAP), to provide an alternative perspective to PCA (Figure 2).
In the following plot, a representation of the UMAP results is provided, where the interaction energy calculated with MLCE has been coloured on a scale from green to blue. The figure shows that, while the body regions of the nanobodies account for regions with a wide range of energy values, the CDR regions are largely composed by representations corresponding to low energy values.

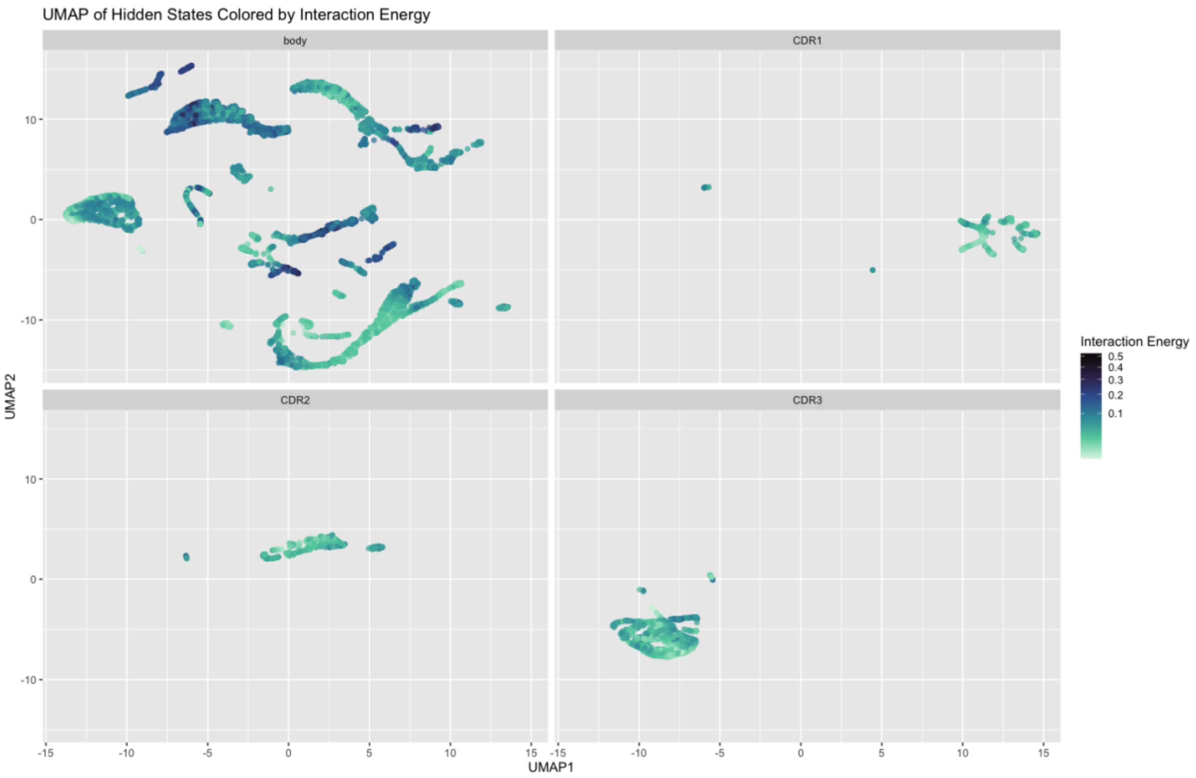

3. Prediction Results: details

3.1 Predicted interacting residues and CDR assignments for the 121 nanobody structures analyzed

In this table, interacting patches predicted by our in-house method MLCE (REBELOT + BEPPE, https://github.com/colombolab/MLCE) are reported. For each of the 121 Nanobodies (Nbs) of our database, we report the PDB ID, the name given by the authors to the Nb, and the residues included in the predicted patches yielded at the end of the pipeline. For clarity, only residues belonging to CDR1, CDR2, or CDR3, predicted by state-of-the-art scheme Chothia using the Maestro Schrodinger suite (see Material and Methods), are reported, even though in most cases also framework residues were included in interacting patches. The only exceptions are 7FBJ_17F6 and 7FBK_20G6, for which Maestro could not find CDRs using the Chothia scheme, and for which we used the prediction of our NanoCDRx method as a reference. Given the high variability of CDR3 sequence and structure, we were most interested in MLCE predictiveness for this loop. For this reason, we considered the prediction successful if at least one CDR3 residue was included in predicted patches. However, CDR1 and CDR2 predictions are also reported to completely assess MLCE performances. Sequences of residues are indicated with the number of the first residue, followed by the one letter code of the residues, and then the number of the last residue (e.g. residues SLLS spanning from residue 104 to 107 are reported as 104SLLS107). The MLCE calculation is performed on the resolved 3D structure of Nbs: for this reason, the numbering of residues always starts from 1, with residue 1 being the first resolved residue in the crystal structure.

PDBID Name	CDR1 residues	CDR2 residues	CDR3 residues	S
6YZ5_H11-D4	26GRTF29	54SGGS57	104SLLS107 – 111TWP113	YES
6ZCZ_H11-H4	25GRTF28	53SGGS56	99HYVSYLLSD107	YES
6ZXN_Ty1	25GF26	53PNSG56	101NL102 – 104SS105	YES
7A29_Sb23	26GFP28	53STGGW57	100VGY102	YES
7B27_NM1230	G26	54AGDL57	102RVGTGER108	YES
7C8V_SR4	26GFP28 – 30YS31	54HGD56	V100	YES
7C8W_MR17	26GF27	--	104LA105	YES
7CAN_MR17-K99Y	26GF27	--	101DGQLAYHYDY110	YES
7D2Z_SR31	29GFP31 – 33WQ34	56SMGYK60	103VG104	YES
7F5G_DL4	27GSFFE31	--	100EGQR103	YES
7F5H_DL28	26GSDFSS31	52SEGS55	D98 – W100	YES
7FAT_Nb1A7	26GYT28 – S31	S53	99GGPSLSYCTGG109 – 11GFL113	YES
7FAU_Nb1B11	25GYT27 – 29SV30	52ASGI55	97GLVRGSCTD105 – Y112 – G114	YES
7FBJ_17F6	29SCA31	--	S88 – 90SAGMCA95	YES
7FBK_20G6	29PCS31	--	90TGDER94	YES
7FG3_K-874	26GS27	55GGT57	100GGDG103	YES
7JVB_Nb20	26GAGA29	51ASGG54	99IETA102	YES
7KGJ_Sb45	G26	54AGQ56	102GHHYE106	YES
7KGK_Sb16	26GFP28 – 30AY31	54YGIK57	--	NO
7KKK_Nb6	25GII27 – 29GR30	52RRGSI56	100ASPAPGD106	YES

7KLW_Sb68	29GSISSI34	56TVNGH60	A103 – 105GY106	YES
7KM5_Nanosota1	26GFT28 – 30KN31	--	98GSKSGHEL105	YES
7KN5_VHHE	26GVTLDY31	--	101GTYYSGNYH109	YES
7KN5_VHHU	25GF26	52SSGG55	99SGSYY103	YES
7KN6_VHHV	26GF27	--	100SLGGWG105	YES
7KN7_VHHW	25GFT27	52SSGD55	101SYYW104	YES
7LX5_WNb10	G26	--	104TWPE107	YES
7LX5_WNb2	25GFTLDY30	53SGGN56	100ATYYSGSYY108	YES
7MDW_Nb105	--	--	103WG104	YES
7MEJ_Nb36	--	--	--	NO
7MFU_Sb14	26GFPVQA31	53STGT56	100VGSS103	YES
7MY2_Nb30	25GLT27	--	--	NO
7MY3_Nb12	G26 – 29HN30	--	100PYFGNSCV107	YES
7N9B_Nb21	--	51ANGGN55	--	NO
7N9C_Nb95	26GR27	--	102VYY104	YES
7N9E_Nb34	25GF26	--	99KDPYGSPWT107	YES
7N9T_Nb17	26GSI28	--	101SAYAP105	YES
7NKT_NM1226	26GSLDY30	52SSGD55	98LQGSYYY104	YES
7NLL_Fu2	26GF27 – D31	54SDG56	100PSFSYTGSTYY110	YES
7OAO_C5	26GVTLGR31	F54	100VTAA103 – S105	YES
7OAP_C1	26GFTNDF31	54SDNT57	101FAG103	YES
7OAP_H3	26GRT28 – 30ST31	54TGSS57	100TIV102	YES
7OAY_F2	26GRT28 – S31	53WSSTP57	101GESYY105 – R108	YES
7OLZ_Re5D06	26GITLD30	--	100PLTKYGSSWY109 – P111	YES
7OLZ_Re9F06	26GRTF29 – N31	53WNSG56	99SDGYL103	YES
7P77_Sb-15	26GFPVKN31	53SGGV56	100VGR102	YES
7Q3Q_VHH-12	26GLT28 – S31	52RWKFGN57	101VG102 – 105IAV107	YES
7Q3R_VHH-F04	25GRA27	53SGGS56	99VDYSGTLTAA108	YES
7Q3R_VHH-G09	26GTGFT30	--	100FDSSDYEVL108	YES
7R4I_Nb2.15	26GYASWAR32	54DFDG57	102GT103	YES
7R4Q_Nb1.29	26GYTINT31	54GSGN57	101YGASGYD107	YES
7R4R_Nb1.10	26GYTYSTC32	53ADG55	99VKDFT103 – T105	YES
7RBY_Nb112	26GLTLDY31	54SDG56	100PSTYYSGTYY109	YES
7TPR_7A3	26GYTSSS31	--	102YNQWG106	YES
7TPR_8A2	--	--	101TYDKYAPCGGFAGTY115	YES
7VNB_n3113	--	--	102GSTG105	YES
7VNE_n3113.1	--	52PSGR55	100SGSTG104	YES

7VOA_aRBD5	26GF27	54SGGI57	101HTVVAGC107 – 112WTDF115	YES
7VQ0_P86	26GR27	--	97DVNGGM102	YES
7W1S_Nb007	25IS26 – 28SSF30	50GIGG53	--	NO
7WD1_R14	26GFTLDY31	54SDG56	100PATYYSGRYYYQ111	YES
7WD2_S43	26GFT28 – Y31	52SSN54	99PDYSGVYYYT108	YES
7WHI_Bn03nano1	26DSS28	53PSG55	102GST104	YES
7WHI_Bn03nano2	D26	53GLGGA57	102FG103	YES
7X2J_Nb70	26GRT28	54DGGT57	100GNQYYSAT107 – S109	YES
7X2L_3-2A2-4	26GSISTL31	52TLDGS56	100GGF102	YES
7X2M_1-2C7	26GD27	53PSGSR57	101PSAHY105	YES
7X4I_aSA3	26GFTSDH31	S53 – 55GNP57	99LWYGR103	YES
7X7E_Nb22	28GGT30	--	102VPPGSRLRGC111 – V113	YES
7Z1A_H11	26GRTFSTA32	54SGGS57	100RVT102 – 104SL105	YES
7Z1B_A10	26GR27 – 30STA32	54SGGS57	100SAT102 – 104SLL106	YES
7Z1C_B5	25GRTFSTA31	53SGGS56	99QAT101 – 103SL104 – 110TWP112	YES
7Z1D_H11-H6	26GRT28 – 30STA32	54SGGS57	100KIT102 – 104SLL106 – 111TWP113	YES
7Z1E_H11-H4_mut	25GRTFST30	R51 – 53SGGS56	101VSYLLSD107	YES
8BEV_W25	26GSIFG30	--	100KNELGF105	YES
8C8P_10D12	26GFT28 – L31	53SGG55	98GLGFGELPP106	YES
8CXN_Nb2-57	--	--	--	NO
8CXQ_Nb1-22	26GRSFNS31	53GSPH56	L102 – 104VGS106	YES
8CY6_Nb2-65	28GTIST32	55NLG57	102LEGGTQ107	YES
8CY7_Nb2-38	26AR27 – S29	--	99GWGIRQP105 – I107	YES
8CY9_Nb1-23	26GRTDSI31	54SGGG57	100SLRVGS105 – S107	YES
8CYA_Nb2-67	G26	53WNGSTR58	103DGVIDGTNANA113	YES
8CYB_Nb1-8	28GRTFSN33	--	102RGSS105	YES
8CYC_Nb2-34	26GRTF29	--	103YSRS106	YES
8CYD_Nb2-45	26GYD28 – 30SI31	52SRVGS56	99IPMTT103	YES
8CYJ_Nb1-25	26GR27	55VG56	103SSS105	YES
8CYJ_Nb2-10	--	--	--	NO
8CYJ_Nb2-62	26GR27	52ARS54 – 56DT57	101VIQYGIVPGND111	YES
8DI5_VHF6	24DF25	--	98SGSG101	YES
8DLX_ab6	G26	--	99WLYGSGY105	YES
8DT8_LM18	25GFTF28	52GSGG55	102YGARDY107	YES
8DT8_Nb136	25GFT27 – 30SS31	52GSGGS56	100GPYDPTDSTY109	YES
8ELO_C4-225	G26	--	103YTYGGSV109	YES
8ELP_C4-240	G26	--	103YDQTGF108	YES

8ELQ_C4-255	26GF27	53GSGGS57	102YYSPYGGP109	YES
8EYG_NbUNK	26GGTFSS31	54DGGA57	101VGKP104	YES
8G72_Nanosota2	26GFN28 – 31TS32	54GY55 – D57	99HNEPYFCDYSG109	YES
8G73_Nanosota3	26GSIFSP31	--	K100	YES
8G75_Nb4	26GF27	54SGGR57	102SRWYCPLQFSAD113	YES
8GZ5_VHH-P17	26GRTSS30	53GNGGT57	--	NO
8H5T_Nb-015	25GFTLDS30	53DG54	--	NO
8H5U_Nb-021	26GTGSTFST33	--	I102	YES
8H91_N19	25GGTFS29	51ADVGF55	98SLQSG102	YES
8HR2_Nb1B5	25GYTYST30	--	98SGW100	YES
8HR2_Nb1C6	26GDTYSS31	54GGDN57	102CPWPDIGTMS111	YES
8K3K_Nb4	29GW30 – E32	--	--	NO
8OWT_A8	26GGT28 – 32TA32	53WRGVR57	100VGNYGL	YES
8OWV_H6	26ESSLAP31	D54 – 56HPTS59	108DS109	YES
8OWW_B5-5	26GSTL29 – N31	54RYGA57	101GPY103	YES
8Q7S_Ma6F06	25GITLDY30	--	98GPLPPGHSCR107 – 109PT110 – 112LG113	YES
8Q7S_Re21H01	27GFT29 – S32	54ITGGS58	104RG105	YES
8Q93_Re21D01	26GFT28 - 30SSF32	52TITGGS57	--	NO
8Q93_Re30H02	26GFTLDY31	53SSDSS57	100PATYYGGNWH109	YES
8Q94_Ma3B12	26GVT28	E52 – 54SSGP57	104HEK106 – 110SPLG113	YES
8Q94_Re32D03	26GITLDY31	--	99GPLPPGISC107 – T111 - 113LG114	YES
8Q95_Ma16B06	26GSI28	52PNSG55	99GVPV102	YES
8Q95_Ma3F05	26GVTLDG31	54SNGP57	104HER106 – S110 – 112LG113	YES
8RBY_Nb1.26	24GYT26 – 29SV30	50PSGRNR55	98SA99 – 101HDPE104	YES
8RJ7_Nb1.29	25GYTINT30	53GSG55	101GASG104	YES
8SK5_VHH-7A9	26GGTAS30	54RNSGSTYV61	105PTLGWY110	YES
8ZER_P2C5	25GYTYC29	--	99CSSGEYL105	YES

3.2 Predictions on Independent Dataset

The following table reports the structural and energetic analysis of seven representative nanobody sequences selected from the INDI database as members of highly divergent clusters. The 3D structures were predicted using the AlphaFold3 Server and subsequently analysed with MLCE to compute residue energy profiles. The table summarises the predicted CDR regions together with the corresponding low-energy uncoupled residues, highlighting that CDR positions consistently correspond to energetically decoupled regions across structurally diverse nanobodies

NbName	Sequence	CDR1 residues	CDR2 residues	CDR3 residues	S
Nano 1	LQLVESGGGLVQSGGSLVLACEVSGFSADNYGIGWFREAPGKSREGVSCINASGGRISYAAFAKGRFNISRENAKNMVYLQVNGLTPEDSATYYCAAGPPRLCTLSVWTVYDYWGQGTQVTVS	25GFSADN30	52ASGGR56	98GPPRL102	YES
Nano 2000000	QLQLVESGGGLVQPGGSLRLSCVATGFFLDDYHIGWFRQAPGKEREVVSCLSSSASGGSANYADSVMGRFTISRDDGRSTVYLQMHSLTSEDTAIYYCAALWSIKAMCPMSSGEYEYWGQGTQVTVS	26GF27	53SSASGGS59	103SIKAMCP109	YES
Nano 3000000	HVQLVESGGGLVQPGGSLRLSCVASGFTFSSYHMSWVRQAPGKGLEWVSSIWSDGRKTHYTTSAKGRFTMSRDDAKNSLYLQLSSLKIEDTGMYYCVKPGLRTETYWGQGTQVTVS	G26	55GR56	99PGLRTET105	YES
Nano 6000000	QLVESGGGLVQPGGSLRLSCAASGFTFGRYYMSLVRQAPGKGLEWVSVIADDGRVTYYADSVKGRFTISRDNAKNTLHLQMNSLKSEDTALYYCATGQSFPYWGQGTQVTVS	24GFT26 - 28GR29	52DGR54	97GQSFPY102	YES
Nano 8000030	LQLVESGGGLVQPGGSLRLSCEASGFRTGHTLDYYSIAWFRQVPGKGREGVSCLANSGENTKYAGSVKARFTIPADTAKTPMYLQMTSRTPEDRAVYYWAAGRVLFRQCRIARVNYDNWGQGTQVTVSS	25GFRTGHT31 - Y34	57SGE59	103RVLFRQ108	YES
Nano 10000030	EVQLVESGGGLVQPGGSLRLSCAASGFTFSSYAMGWYRQAPGKQRELVATITSGDSTNYADSVKGRFTISRDNAENTMYLQMNSLKHEEDTALYYCATDPVRGGRYGLDYWGKGTQVTVSS	26GF27	53SGD55	99DPVRGGRYG107 - D109	YES
Nano 11228352	VQLVESGGGLVQSGGSLTLSCVASGAYFILALGWYRQAPGKRRELVAVITYAGAINYGSFAQGRFTISRDNTKKTVYLQMNNLKPEDTAIYYCNARPIVEASNNYWGQGTQVTVSS	25GAYFSIL31	52YAGA55	97RPIVEASN104	YES

4. Counterfactual Analyses: modified sequences

4.1 Shifted and randomised 7C8V_SR4 and 8ELO_C4 nanobody's sequences

Two representative nanobodies (7C8V and 8ELO_C4) were used as template to generate modified sequences by:
- shifting the native CDRs to three different random, unnatural positions along the sequence scaffold (7C8V_SR4_shifted01, 7C8V_SR4_shifted02, 7C8V_SR4_shifted03; 8ELO_C4-225_shifted01, 8ELO_C4-225_shifted02, 8ELO_C4-225_shifted03)
- completely randomising the entire sequence (7C8V_SR4_randomised, 8ELO_C4-225_randomised)

7C8V_SR4 – original sequence
QVQLVESGGGLVQAGGSLRLSCAASGFPVYSWNMWWYRQAPGKEREWVAAIESHGDSTRYADSVKGRFTISRDNAKNTVYL
QMNSLKPEDTAVYYCYVWVGHTYYGQGTQVTVSAGRAG
7C8V_SR4_shifted01
QVQLVESGGGLGFPVYSWVQAGGSLRLSCAASNMWWYRQAPGKEESHGDSEWVAAITRYADSVKGRFTISRDWVGHTYNAK
NTVYLQMNSLKPEDTAVYYCYVYGQGTQVTVSAGRAG
7C8V_SR4_shifted02
QVQLGFPVYSWVESGGGLVQAGGSLRLESHGDSSCAASNMWWYRQAPGKEREWVAAITRYADSVKGRFTISRDNAKNTVYL
WVGHTYQMNSLKPEDTAVYYCYVYGQGTQVTVSAGRAG
7C8V_SR4_shifted03
QVQLVESGGGLVQAGGSLRLSCAASNMWWYGFPVYSWRQAPGKEREWVAAITRYADSVKGRESHGDSFTISRDNAKNTVYL
QMNSLKPEDTAVYYCYVYGQGTQWVGHTYVTVSAGRAG
7C8V_SR4_randomised
VAQYEGHQTAAYRKPLSGGAFSTSLRVWSFICGVYQDSEYYTRCWGRVGGGQNVWNSSAEMRGLQGRMWAQPSDSANLRV
TQWIYDGNAKESGATVSLAVGTEAYVYYVVTLKHDPGK
8ELO_C4-225 – original sequence
EVQLQESGGGLVQPGGSLRLSCAASGFTFSSYAMGWYRQAPGKEREWVCAISGSGGSTYYADSVKGRFTCSRDNSKNTLYL
QMNSLKPEDTAVYYCARGSFYYTYGGSVGFDAFDYWGQGTQVTVS
8ELO_C4-225_shifted01
EVQLQESGGGLVQPGGSLRLGFTFSSYSCAASAMGSGSGGSWYRQAPGKEREWVCAITYYADSVKGRFTCSRDNSKNTLYL
GSFYYTYGGSVGFDAFDYQMNSLKPEDTAVYYCARWGQGTQVTVS
8ELO_C4-225_shifted02
EVQLQESGGGLGFTFSSYVQPGGSLRLSCSGSGGSAASAMGWYRQAPGKEREWVCAITYYADSVKGRFTGSFYYTYGGSVG
FDAFDYCSRDNSKNTLYLQMNSLKPEDTAVYYCARWGQGTQVTVS
8ELO_C4-225_shifted03
EVQLQESGGGLVQPGGSLRLSCAASAMGWYRQGFTFSSYPGKEREWVCAITYYSGSGGSADSVKGRFTCSRDNSKNTLYLQ
MNSLKPEDTAVYYCARWGQGTQGSFYYTYGGSVGFDAFDYTVS
8ELO_C4-225_randomised
LFGTGVGLWIQTYADQNGGYQRTRVESEYRQWYSYSYFQPGGFGALWVSVLSYSPAMVEQASCNATKTGYSKSGGGGYSTC
GQPGASMRFVVLATCSASLDRSLCRFEDYYKDSNGSTDKEGFVG

4.2 Shifted and randomized nanobody sequences from 7C8V_SR4 and 8ELO_C4 predicted by NanoCDR-X

Predictions generated by nanocdr-x on the artificially modified sequences. Predicted CDR1 regions are highlighted in blue, CDR2 in orange, and CDR3 in green. The boxed regions indicate the positions where the original CDRs have been placed (shifted) within the sequence. The order of the sequences for each nanobody is: _shifted01, _shifted02, _shifted03 and randomised.

7C8V_SR4

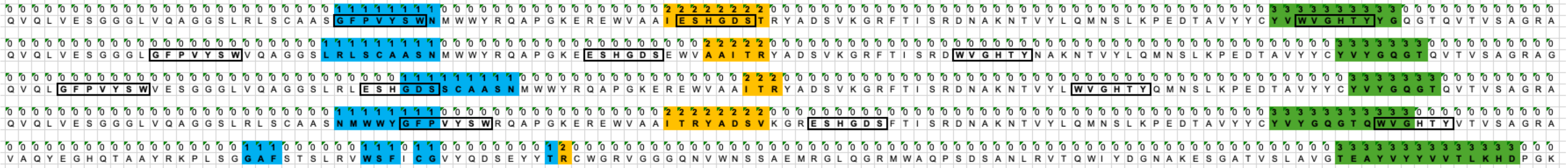

8ELO_C4

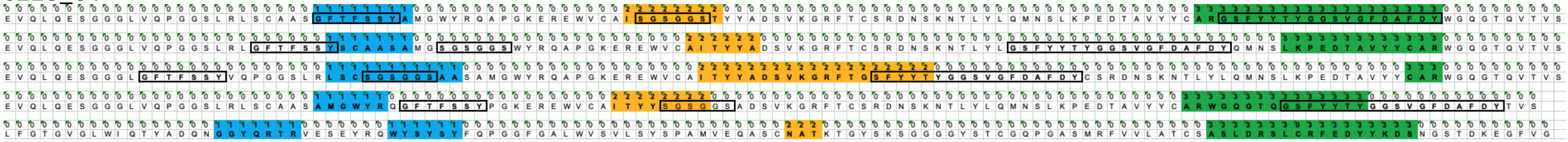

4.3 Shifted and randomized nanobody sequences from 7C8V_SR4 and 8ELO_C4 predicted by the MLCE.

Description of the file reporting the structural and energetic analysis of the artificially modified nanobody sequences. The 3D structures of the shifted and fully randomised sequences were predicted using the AlphaFold3 Server and subsequently analysed with MLCE. The table summarises the predicted structures together with the corresponding MLCE energy profiles, highlighting the multiple uncoupled low-energy regions identified in these highly disordered and loop-rich conformations

identifier	nanocdr-x predicted CDR1	nanocdr-x predicted CDR2	nanocdr-x predicted CDR3	MLCE predicted CDR1	MLCE predicted CDR2	MLCE predicted CDR3	Notes
7C8V_SR4	GFPVYSWN	IESHGDST	YVWVGHTYYG	GFP-YS	HGD	V	green-highlighted cells indicate that the sequence reported correspond to structural loop
7C8V_SR4 _shifted01	LRLSCAASN	AAITR	YVYGQGT	-	-	-	
7C8V_SR4 _shifted02	GDSSCAASN	ITR	YVYGQGT	DSCAA	-	-	MLCE does not directly classify weakly coupled regions as CDRs 1, 2 or 3 --> for shifted and randomized structures, only residues corresponding to nanocdrx predictions also found by MLCE are reported
7C8V_SR4 _shifted03	NMWWYGFP	ITRYADSV	YVYGQGTQWVG	NM	-	GQGT	MLCE often finds uncoupled regions different from the CDRs found by nanocdrx, because the prediction is performed on the 3D structure of the shifted or randomized structure, which are artificial and thus full of loops (loops are often energetically uncoupled

							from the rest of the structure)
7C8V_SR4_randomised	GAF	R	TEAYVYYVVTLKHD	-	R	-	
8ELO_C4-225	GFTFSSYA	ISGSGGST	ARGSFYYTYGGSVGFDAFDY	G26	-	YTYGGSV	
8ELO_C4-225_shifted01	YSCAASA	AITYYA	LKPEDTAVYYCAR	YSCAASA	-	-	
8ELO_C4-225_shifted02	LSCSGSGGSAA	ITYYADSVKGRFTGSFYYT	CAR	GG	ADS-KGRFTGSFYYT	-	
8ELO_C4-225_shifted03	AMGWYR	ITYYSGSG	ARWGQGTQGSFYYTY	A-GWY	YSGS	GQGTQGSFYYTY	
8ELO_C4-225_randomised	GGYQRTR	NAT	ASLDRSL	GGYQ	-	-	orange-highlighted cells indicate that the sequence reported do not correspond to structural loop

5. Benchmarking

5.1 Comparisons with other deep-learning approaches

The following table compares nanocdr-x with other state-of-the-art deep learning approaches commonly used in antibody and nanobody analysis. The table summarises each tool according to its primary computational task, required input format, and type of output produced. In particular, it highlights the methodological differences between sequence labelling tools, protein language models, and structure prediction methods, clarifying why a direct quantitative benchmark is not feasible and illustrating the distinct niche occupied by nanocdr-x as a direct sequence-to-label predictor.

Tool	Type / Main Task	Input	Output	Can perform direct CDR labeling?	Reason for exclusion from benchmark (F1 assessment)
**nanocdr-x**	Sequence Labeling (CDR identification)	Raw nanobody sequence	CDR sequence classification (body, CDR1, CDR2, CDR3)	Yes	N/A
**NanoBERT**	Protein Language Model, Masked Language modelling, Embeddings	Nanobody sequence	Embeddings, Masked sequence prediction	No	Requires training a downstream classifier (fine-tuning).
**AntiBERTy**	Protein Language Model, Masked Language modelling, Embeddings	Antibody sequence	Embeddings, Masked sequence prediction	No	Outputs embeddings; not residue-level labels.
**ProtBERT**	Protein Language Model, Masked Language modelling, Embeddings	Protein sequence	Embeddings, Masked sequence prediction	No	Requires training a downstream classifier (fine-tuning).
**simpleDH3**	Structure Prediction	Antibody sequence	3D Coordinates (PDB)	No	Predicts geometry, not labels. Requires external numbering tool to define CDRs.
**ABlooper**	Structure Prediction	Heavy and Light Chain sequence (numbered)	3D Coordinates (PDB)	No	Requires paired chains (H+L) or pre-numbered input; does not support raw single-chain discovery.

**AntiBERTa**	Protein Language Model, Masked Language modelling, Embeddings	Antibody sequence	Embeddings, Masked sequence prediction	No	Outputs embeddings; not residue-level labels.
**AlphaFold3**	Structure Prediction	Protein Sequence	3D Coordinates (PDB)	No	Predicts geometry, not labels. Requires external numbering tool to define CDRs.
